# Quantitative approaches to energy and glucose homeostasis: machine learning and modelling for precision understanding and prediction

**DOI:** 10.1098/rsif.2017.0736

**Published:** 2018-01-24

**Authors:** Thomas McGrath, Kevin G. Murphy, Nick S. Jones

**Affiliations:** 1 Department of Mathematics, Imperial College, London SW7 2AZ, UK; 2 Department of Medicine, Imperial College, London SW7 2AZ, UK; 3 EPSRC Centre for Mathematics of Precision Healthcare, Imperial College, London SW7 2AZ, UK

**Keywords:** energy homeostasis, mathematical biology, machine learning, glucostasis

## Abstract

Obesity is a major global public health problem. Understanding how energy homeostasis is regulated, and can become dysregulated, is crucial for developing new treatments for obesity. Detailed recording of individual behaviour and new imaging modalities offer the prospect of medically relevant models of energy homeostasis that are both understandable and individually predictive. The profusion of data from these sources has led to an interest in applying machine learning techniques to gain insight from these large, relatively unstructured datasets. We review both physiological models and machine learning results across a diverse range of applications in energy homeostasis, and highlight how modelling and machine learning can work together to improve predictive ability. We collect quantitative details in a comprehensive mathematical supplement. We also discuss the prospects of forecasting homeostatic behaviour and stress the importance of characterizing stochasticity within and between individuals in order to provide practical, tailored forecasts and guidance to combat the spread of obesity.

## 1. Introduction

The growing crises of obesity and metabolic syndrome can be viewed as failures of energy homeostasis: our regulatory systems are poorly adapted to deal with the availability of appetizing high-calorie foods. Although the trend of increasing bodyweight has been continuing for decades, in recent years new data sources have become available that may transform the way we research and treat obesity. Examples of these data sources include wearable technology such as activity monitors and continuous glucose measuring devices, activity and food logging apps as well as an impressive range of technologies for monitoring neuronal activity *in vivo*. Although these technologies differ substantially in their sophistication and intended uses, they share one key feature: the production of orders of magnitude more quantitative data than previous techniques. For instance, a glucose monitoring device may collect a measurement every 5 min, generating hundreds of data points per day compared to two or three measurements taken daily by a typical diabetic. Connected food and activity logging apps can leverage large databases to report detailed information about the nutritional contents of a meal given only a barcode, and can generate energy expenditure figures personalized to a user's weight, age and gender. Two-photon imaging can give exquisitely detailed information into how the firing of specific neuronal populations drives feeding behaviour, generating many parallel time series of neuronal firing [1].

This explosion of data creates opportunities, but only if the relatively unstructured data can be parsed for understanding and prediction. A traditional approach to large amounts of quantitative data has been to fit a tailored mathematical model, either derived empirically or using a physiological understanding of the system involved. In recent years, advances in machine learning have opened up a new way of understanding these datasets. This review covers both model-based and machine learning approaches to understanding energy homeostasis, as well as developments on the cutting edge, where models are being integrated into machine learning tools to further improve prediction. One of the key sources of innovation thus far has been research into understanding and control of glucostasis, spurred by the desire to engineer an artificial pancreas. This review, therefore, looks first at the progress made modelling on our understanding of glucostasis because the state of the art is more advanced in this field and the techniques employed can serve as a model for use elsewhere.

We also discuss the need for personalization in models, particularly if they are to be used to guide behavioural interventions. Given the wide inter-individual variation in glucose response following a meal [1], it is highly likely that inter-individual variation plays a significant role in other homeostatic processes. If we fail to account for this, models intended to optimize treatments will perform sub-optimally or fail as they are poorly adapted to the individual being treated. For this reason, we discuss approaches to model personalization throughout the review by either reviewing successful examples, or suggesting pathways towards individualizing current models table 1 (box 1 and box 2).

**Table 1.** Summary of review contents. This review covers diverse but connected (figure 1) aspects of energy homeostasis. This table is intended to serve as a quick overview and guide to the phenomena and models we discuss.

physiological problem	methods	data sources	section	electronic supplementary material sections
*Endocrine mechanisms* (§2)				
endocrine regulation of blood glucose	differential equations	plasma metabolite and hormone concentrations	2.1	(S2.1–S2.4)
blood glucose dynamics after eating	differential equations	stomach fullness and circulating metabolites	2.1	(S2.5–S2.7)
inter-individual variation in glucostasis	machine learning	patient-specific behavioural data (e.g. sleep duration), metabolites	2.2	(S2.8–S2.10), box 1
emergence of diabetes and leptin resistance	multiscale modelling	circulating metabolites, pancreatic β cell mass	2.3	(S2.11–S2.14)
*Body composition* (§3)				
changes in body weight and composition	differential equations	average food intake, body weight and composition	3.1	(S3.1–S3.7), box 2
effect of macronutrient intake on growth and development	differential equations	growth curves, body composition measurements, energy intake/expenditure	3.2	(S3.8)
*Feeding behaviour* (§4)				
food intake within a meal	control theory	feeding time series	4.1	(S4.1)
endocrine regulation of food intake	differential equations	food intake, circulating hormone concentrations	4.1	(S4.2, S4.3)
food intake planning	control theory	feeding time series	4.1	(S4.5, S4.6)
learning the rules governing behaviour	machine learning	feeding time series, neuronal activity	4.2	(S4.7, S4.8)

**Figure 1.**
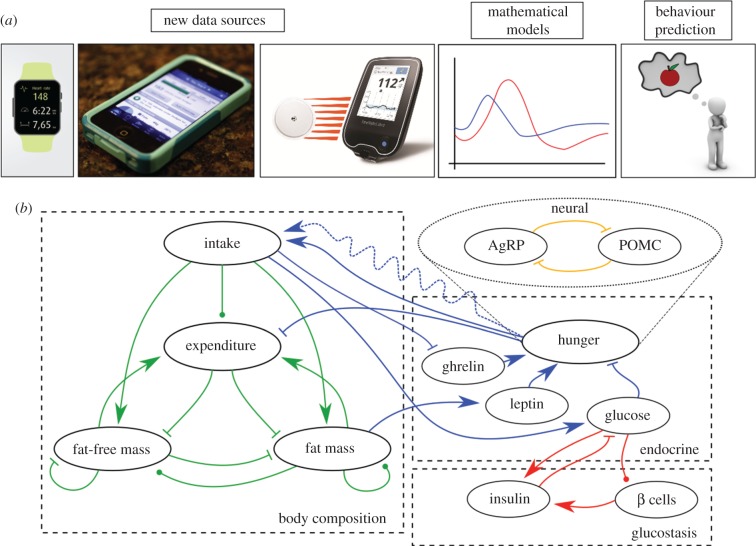
New data sources need new modelling techniques to maximize their predictive ability. In particular, we can now work towards understanding the roles of inter-individual variation and stochasticity because of the finer temporal resolution allowed by personal omics devices (*a*). These can be fed into traditional physiological models, summarized in (*b*), to understand how observed feeding behaviour affects internal state, for example, blood glucose or endocrine levels. The state of the art across the literature is summarized here: each model in this review contains a subset of these entities and connections. Lines with arrowheads indicate positive effects, bar ends denote negative effects and circular ends can be positive or negative. Glucostatic models (red lines, §2) investigate the dynamics of glucose, insulin and pancreatic β cells in response to glucose infusion. Endocrine models (blue lines, §2) are a relatively recent development, and model how endocrine mechanisms mediate energy intake and expenditure. Energy balance models (green lines, §3) consider the distribution of calories within the body, but do not typically predict intake or expenditure. The link between physiological state and behaviour is often considered through the perspective of control theory (§4), although stochastic control policies (represented by the dashed line) have not received sufficient attention, leading to poor predictive ability.

Box 1. **Combining machine learning and model-based techniques for large datasets.**

Machine learning is a broad label that is applied to a range of statistical prediction techniques, often using large quantities of data and relatively flexible predictive models. In a machine learning problem we typically have one or more outcomes we want to predict, as well as a set of data associated with each outcome. A concrete example for this might be predicting blood glucose level 30 min after a meal. Available data might include blood glucose levels at 5 min intervals preceding the meal, meal size and macronutrient composition. Each of these corresponds to some numerical value, so we intend to predict a single unknown variable (future glucose concentration) with a vector of measurements (past glucose levels, meal data). The known data are referred to as features or explanatory variables. Typically, we would then choose a statistical model with some unknown parameters *θ*, which we train on data where we know both the explanatory variables and the ‘predicted’ variable. Training corresponds to finding the values of *θ* that best explain the known data. For instance, in linear regression, this means finding the slope and intercept. The ‘trained’ model can now be used to predict future outcomes for which we only know the explanatory variables. A problem very similar to the example given above was solved recently using boosted decision trees [1], which are in effect an extremely large bank of yes/no questions regarding the data, leading to accurate predictions and the ability to tailor diets to individuals based on personal information such as microbiome sequencing.

In the blood glucose prediction example above, only untransformed data were used. An important technique in machine learning is generating new features that will increase the accuracy of our predictions. This is known as feature engineering. This review presents a wide array of techniques for transforming one set of observations into another. Decades of biological experience are contained within these models, which can obtain hard to measure quantities from easily observable ones, for instance, converting meal data into expected blood glucose and insulin concentrations. This wealth of biological knowledge has yet to be put to significant use for making predictions, but could have a huge impact; it is likely that apparently unpredictable behaviour may be driven by underlying explanatory variables (figure 4) that we simply cannot determine from easily observable data. Feature engineering using models, for instance those presented in this review, could allow access to these otherwise hidden explanatory variables in an interpretable way. We have not discussed the specifics of individual models in this box, and instead refer the interested reader to the supplement for details of models in this paper, or to the many excellent textbooks available [2–5].

Box 2. **Dynamical systems and homeostasis.**

In this review, we have made use of concepts from the theory of dynamical systems. In this box, we provide a brief qualitative overview of terms used elsewhere in the article. A dynamical system is defined as a set of variables and functions that govern how these variables change through time given the current value of each variable. The set of all possible values of all of the variables is referred to as phase space, a point in phase space represents the state of a system, and the path that is taken by a system through phase space is called its trajectory or flow. The number of variables that comprise the system is known as its dimension; a one- or two-dimensional system can have its phase space represented as a diagram (known as a phase portrait) as described in the examples below.

A system may possess points in phase space which a system will never leave once it has arrived at them; these are called fixed points. For example, a ball rolling in a valley will eventually come to rest at the bottom of the valley, which is the fixed point of the system. Similarly, a ball at rest on the flat top of a hill will, without perturbation, never roll down. These two fixed situations illustrate two important kinds of fixed points: stable fixed points (the valley) and unstable fixed points (the hill). More complicated systems can also possess limit cycles—fixed orbits in phase space. We expect that a perfect homeostatic system should possess stable fixed points or limit cycles; this corresponds to our intuition that the system will return to either a stable state or a stable oscillation (in systems in which, for example, circadian rhythms are important).

The stability of a system given by a set of equations can vary based on the parametrization of that series of equations. For example, the generic form of a quadratic equation is given by *ax*^2^ + *bx* + *c* = 0. In this equation *a*, *b* and *c* are the parameters and *x* is the variable. A change in stability brought on by a change in parameter value is known as a bifurcation. Bifurcations have already been encountered in endocrine modelling, for example, as described in §4.

In systems with multiple variables, there may be lines in phase space along which a particular variable does not change. These are referred to as nullclines and are of great importance in determining the stability of a system. Variables may change with different characteristic speeds in a system; for example, changes in insulin secretion take place on a much faster timescale than changes in adiposity. In such systems it can be useful to introduce the concept of fast and slow manifolds, corresponding to the behaviour of the system on different timescales. One approach to multiscale systems is to split them into multiple subsystems, each functioning on different timescales. The behaviour of one subsystem can manifest itself in another subsystem through a change in parameter values. Dynamical systems theory thus offers many tools for the analysis of time-evolving biological systems, and the interested reader is directed to excellent texts by Strogatz or Kaplan & Glass [6,7] for a more detailed introduction.

## 2. The biology of energy homeostasis

### 2.1. Regulation of glucose and fatty acid metabolism

In this section, we provide a brief overview of the most important elements of human metabolism to provide context and motivation for the models that follow (§2). This is a short overview of a deep and extensively studied area, and readers are directed to other resources for more detail [8]. Energy homeostasis at the level of metabolic fluxes is primarily governed by endocrine mechanisms. These can store surplus circulating metabolites in tissues when supply exceeds demand, or mobilize stored energy during times of need, for instance, during exercise. Long-term energy storage is accomplished by fats, whereas short-term requirements are typically satisfied by carbohydrates. Glycogen can be used more rapidly, whereas triacylglycerol must be metabolized into fatty acids before it can be used. There are multiple depots of both fat and carbohydrate in the body; the most important fat stores are in adipose tissue, skeletal muscle and the liver [9]. The majority of stored fat is held as triacylglycerols; however, these are unsuitable for transport in the blood as they are almost insoluble in water. Thus, they must be converted into a simpler form (non-esterified fatty acids) in order to be transported). The metabolic fluxes involved in fat storage have received relatively little mathematical study. Carbohydrate fluxes, on the other hand, have been extensively modelled, in part due to interest in understanding the causes and progression of diabetes. Glycogen is the primary carbohydrate store in mammals, with the major glycogen depots found in the liver and skeletal muscle [8]. Glycogen can be converted into glucose and then transported via the bloodstream when energy requirements increase, a process which is promoted by glucagon. The storage of excess glucose as glycogen is promoted by insulin when energy supply exceeds demand, for instance, following a meal. Insulin is also a key regulator of fat storage, as it promotes fat storage in adipose tissue and suppresses its mobilization. The importance of insulin in metabolic control makes it an important object of study, and it has received a great deal of mathematical attention (§2).

These feedback loops can break down, however. One key way that this can happen is the development of resistance to insulin or leptin. Insulin resistance is the failure of insulin secretion to lead to the deposition of circulating glucose, which may occur due to a variety of causes [10], with the accumulation of fatty acids in cells being an important cause linking increases in adiposity and the development of diabetes. Failure of insulin action means that circulating glucose remains high, stimulating the secretion of more insulin. This has the potential to lead to damage to the pancreatic β cells responsible for insulin secretion, as we discuss in §2.

### 2.2. Endocrine, interoceptive and neuronal regulation of satiety

In addition to being a key player in the regulation of metabolic fluxes, insulin also has a strong effect on feeding behaviour via receptors in the hypothalamus [11]. This brain area is a powerful regulator of feeding behaviour, and integrates other endocrine signals such as leptin. Leptin is released from adipose tissue, and acts to suppress food intake. Integration of endocrine signals is accomplished via neurons in the arcuate nucleus. Similarly to insulin resistance, leptin resistance can also occur via a number of pathways, but is broadly defined as the failure of raised leptin levels to decrease food intake [12]. One key mechanism is alteration of leptin receptor signalling [13], decreasing the effect of leptin once it reaches the brain, particularly in the arcuate nucleus. Leptin resistance can also occur via a decrease in the ability of leptin to cross the blood–brain barrier [14]. In this case, although leptin concentration in the periphery is high, less of this leptin can have its effect in the brain. Both of these effects have been considered by a model of leptin resistance as discussed in §3. Two of the most important populations are those expressing agouti-related peptide (AGRP) and those that express pro-opiomelanocortin (POMC). The balance between AGRP and POMC activity leads to stimulation or suppression of food intake. Other signals from the body are also integrated in the brain to control feeding: the gut–brain axis modulates feeding via endocrine mechanisms such as ghrelin and cholecystokinin and through direct neural signals, for instance, of gut distension [15]. Gut distension and other interoceptive cues also affect other brain areas including the parabrachial nucleus [16]. The powerful control loop between endocrine signalling altering food intake and in turn being altered by the results of feeding makes this an appealing target for modelling, but the complexity of the system presents a substantial challenge. Progress on this problem is collected in §4. Experimental evidence has also suggested a major role for learning and reward in the control of food intake [17], and that AGRP neurons transmit a teaching signal [18]. This has yet to be explored mathematically, although a ready-made framework is available in the form of reinforcement learning, which we discuss in §4. This brief introduction to neural and endocrine control has only covered the basics of a rapidly expanding field, which has been extensively reviewed elsewhere [15,19,20].

## 3. Models of endocrine feedback provide a physiological basis for understanding energy homeostasis

Mathematical models of glucostasis have a long history, and were originally devised to model the response to the intravenous glucose tolerance test and produce a measure of insulin sensitivity. Glucostatic models largely use ordinary differential equations (ODEs) with multiple compartments representing different parts of the body. In these, the rate of flow from one compartment to another (for example, of glucose from the stomach contents to the blood) is given by a set of equations. Solving these gives time courses for the compartments, for instance, blood glucose over time.

There has been a long-running attempt to create an artificial pancreas for type 1 diabetics [21,22]. This is an inherently model-driven exercise: to deliver a bolus of the insulin at the correct time, the artificial pancreas must have some idea of how this will affect blood glucose in the future, leading to a continued interest in models of glucostasis relevant to more realistic situations than the intravenous glucose tolerance test. These efforts have begun to show fruit, leading to a simulation model approved for preclinical testing of insulin delivery algorithms [23,24] and closed-loop insulin pumps now being brought to market.

In what will become a recurring theme, the main challenge in taking this technology further is that of variation. There are several components of variation: inter-individual, predictable inter-event (e.g. due to diurnal changes), random variation (where no cause can be identified) and measurement error. A source of variation can be a member of several of these categories simultaneously, for instance, different individuals appear to have different circadian variation in insulin sensitivity [25]. Quantifying these sources of variation is a key requirement to make prediction of glucose variation as accurate as possible on an individual basis.

### 3.1. Pancreatic secretion of insulin in response to a glucose challenge has been accurately modelled

Insulin has a well characterized, and critical, role in the regulation of glucose homeostasis, and has been extensively studied both physiologically and mathematically. The short timescale of insulin action in response to a meal has made it an ideal candidate for mathematical modelling, as it allows the predictions of a model to be easily tested in controlled conditions. In response to elevated blood glucose, insulin is secreted from the β cells in the pancreas to modulate glucose levels, energy storage and appetitive behaviour [26,27]. Insulin is key to glucostasis—the maintenance of blood glucose at a certain level. At its most basic, this can be modelled by a set of coupled differential equations expressing insulin levels as a function of insulin secretion and clearance, and glucose levels as a function of glucose arrival and glucose clearance (due to both insulin-dependent and insulin-independent processes) [28,29] (electronic supplementary material, S2.1, S2.2). The balance between these two functions will define a stable equilibrium point to which the system will return following a perturbation, as would follow ingestion of a meal.

A thorough review of glucostatic models has been carried out by Pattaranit & van den Berg [30], who consider developments from this simple two-variable ordinary differential equation (ODE) model to more complex models incorporating delays and extensions to take into account additional metabolic and endocrine components such as glucagon and non-esterized fatty acids. Incorporating delays captures the time necessary for secretion of additional insulin in response to elevated glucose and the time taken for it to effect glucose clearance, and has been analysed by a number of researchers [31–33] (electronic supplementary material, S2.3). Furthermore, the original minimal model does not model glucose before it has entered the bloodstream and after its exit; these terms are simply treated as a source and a sink, respectively: glucose outside of the circulation is ignored. To study the onset of obesity it is necessary to keep track of the clearance of glucose in ways pertinent to the generation and growth of adipose tissue. A model of this kind was developed by Roy & Parker by considering the creation of non-esterified fatty acids [34] (electronic supplementary material, S2.4). Given the important role non-esterified fatty acids play in metabolism (see §1), more development of this model may be useful.

Models of glucostasis in response to a bolus of glucose are useful for understanding the response to intravenous glucose tolerance tests, but insufficient for understanding glucostasis in response to meals. To consider this more realistic situation we need an understanding of how meals are processed by the digestive tract and lead to glucose arrival into the bloodstream. Several models of the digestive system have been formulated, typically in the form of multiple-compartment ODEs where the compartments represent parts of the stomach. The most commonly used model is a multiple-compartment nonlinear ODE model [35,36] (electronic supplementary material, S2.5), although other models have been suggested [37,38] (electronic supplementary material, S2.6 and S2.7). Given the substantial degree of stable inter-individual variation in gastric emptying [39], and the effect of meal composition [40,41] a model that allows for prediction of gastric emptying rate for an arbitrary meal or individual will be an important component of personalized approaches to combating obesity and diabetes.

#### 3.1.1. Statistics and machine learning in glucostasis

Applications of machine learning to problems in energy homeostasis are most advanced in the modelling of glucostasis, which we review in this subsection. Attempts to control glucostasis have largely been driven by the goal of engineering an artificial pancreas and managing its insulin delivery to aid with the management of type 1 diabetes mellitus. Until recently, most approaches were based on using physiological models similar to those outlined above to predict the future course of blood glucose and choose insulin delivery times that minimized the risk of hypo- or hyperglycaemic events. Prediction of blood glucose outside of controlled laboratory conditions is complicated by the fact that multiple complex systems are working simultaneously to control blood glucose, which is, in turn, being perturbed by the absorption of glucose from the digestive tract. Experimental data to calibrate these models have been derived by use of tracer techniques and deconvolution in order to determine time-courses for each model variable [21]. This allows for models of each system to be validated and parametrized independently, but is time-consuming and experimentally challenging. This presents issues for individualization as inter-individual variation must be accounted for by tracer measurements and parameter fitting for each patient. Furthermore, sources of dynamic but predictable intra-individual variation, such as sleep quality [42,43], digestive tract emptying rate [39,44] and time of day [45], lead to an unmanageable growth of experimental measurements. One resolution to this issue is to exploit our knowledge of how these external, easily observed factors affect glucostasis by incorporating them as explanatory factors in a mixed effects model. This approach to individualization has been applied in the context of intravenous and oral glucose tolerance test data with several demographic variables including age, height, weight and sex [46,47].

There is currently a great diversity of machine learning methods (see box 2) in use, both aimed specifically at individualization [48] and at wider applications in diabetes research [49]. Models of this type typically take in important explanatory variables that affect glucose homeostasis but are easily available, such as historic glucose data from continuous glucose monitors, feeding data and exercise information. Conventionally, these would be used directly in one or more of the deterministic models described to predict future blood glucose concentrations and allow an artificial pancreas to release insulin accordingly. Alternative approaches (as described in [48]) are to either learn to predict future blood glucose values from the observed data directly, or to derive new time data from the observed data using deterministic models and then learn to predict using both the original and model-derived data. This last approach, known as feature engineering, can increase predictive accuracy [50,51]. There is a wide diversity of predictive models in use, including neural networks (electronic supplementary material, S2.8), time-series models (electronic supplementary material, S2.9) and random forests (electronic supplementary material, S2.10). The majority of models evaluated aim to predict blood glucose concentration on timescales of minutes or hours, and are evaluated with least-squares error against the true data. Given the range of models and similar predictive goals, a very useful project would be to compare predictive performance of each model on a single dataset, as it is currently unclear how the performance of these models compares. Model evaluations like this have been extremely successful in driving progress in computer vision, for instance, the popular annual ImageNet competition, and a similar blood glucose prediction competition could advance the state of the art dramatically. If pre-existing datasets could be pooled this would also overcome the relatively small sample sizes in much of the work to date, and reduce the barrier to entry for researchers without the ability to collect clinical data. Although this would increase the diversity of populations in the dataset, this is a challenge that these algorithms will have to meet when deployed in real clinical usage.

#### 3.1.2. Multiscale models of endocrine systems predict aetiology of regulatory disorders

**Figure 2.**
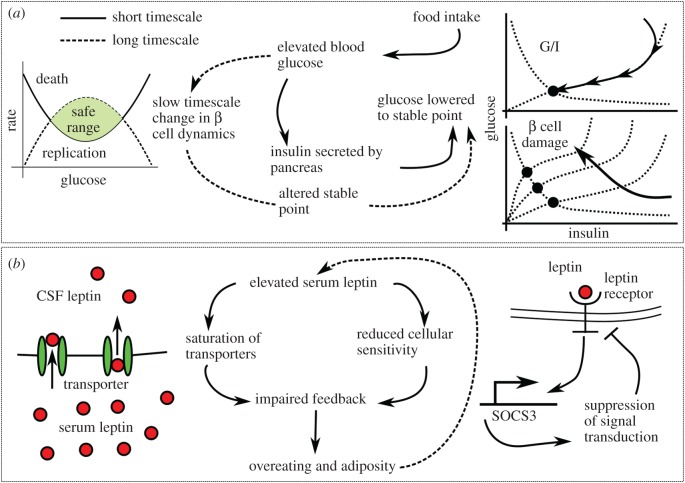
(*a*) Dynamical systems models of glucostasis illustrate the importance of considering both short- and long-term behaviour. The schematic on the left illustrates the interplay between short-term glucostasis due to the action of insulin and the long-term effect of elevated glucose on the β cells in the pancreas. Initially, the glucose/insulin system is at a fixed point: glucose and insulin concentrations are stable. After receiving a glucose spike, for instance following a meal, the system evolves towards a new set point at a higher glucose concentration. Glucose levels above a certain level lead to pancreatic β cell death (shaded region) and the amount of time the system spends in this region, as well as the amount glucose levels exceed the threshold, determine the level of β cell damage. This damage reduces insulin secretion, which in turn moves the fixed point to a new value. The degree to which this movement occurs in a single cycle has been exaggerated to increase the clarity of the figure. (*b*) A similar model of leptin resistance, in which leptin receptor density depends nonlinearly on leptin concentration, also shows a rich phenomenology. As the effect of leptin concentration on food intake and the rate at which excess leptin concentration causes receptor desensitization are varied (as can happen when exposed to more palatable food and during ageing, respectively), the steady state of the system can vary sharply. A mouse with initial low body fat will return to a healthy steady state, whereas an obese one will return to obesity following a perturbation.

The models of insulin-mediated glucose homeostasis discussed in the previous subsections treat the ability of the body to secrete insulin from the β cells of the pancreas in response to glucose levels as fixed. However, in reality a damaged pancreas may be less able to secrete insulin in order to match the demands placed upon it by elevated glucose levels, leading to a higher steady-state blood glucose concentration. Blood glucose may also have a nonlinear effect on pancreatic β cell mass, with moderately elevated levels leading to β cell proliferation and highly elevated levels resulting in loss of β cells due to apoptosis [52,53]. The interplay of the short timescale insulin–glucose system with the long timescale dynamics of pancreatic β cell mass has been investigated mathematically, which we summarize in figure 2*a*, with the results suggesting multiple pathways to diabetes.

Topp *et al*. [54] were the first to couple insulin regulation with β cell mass in a key early result (electronic supplementary material, S2.11). By combining models of insulin-mediated glucostasis [29,55] with a nonlinear model of pancreatic β cell mass [56] they obtained results for the dynamical structure of the composite system. The interplay of the long-term changes in β cell mass and baseline glucose concentration leads to complex and medically relevant dynamics: for glucose concentrations below a certain threshold, the system is attracted to a stable fixed point where both glucose and pancreatic β cell mass are maintained at a healthy level. The system possesses a saddle point, however, and upon moving past the saddle point on the slow manifold, β cell mass tends towards zero, leading to high levels of blood glucose. Further developments [57,58] (electronic supplementary material, S2.12) led to a multiscale model of glucose homeostasis that considers the impact of glucose arrival patterns [59] (electronic supplementary material, S2.13). Spikes in glucose arrival are predicted to lead to worse outcomes as they cause blood glucose levels to spend more time at concentrations leading to β cell damage. This illustrates the importance of considering glucose arrival, and thus of modelling the gut (see above).

Finally, a recent model by Jacquier *et al*. [60] performs a dynamical systems analysis of a model of progressive leptin resistance coupled to the energy partition model of Hall *et al*. (electronic supplementary material, S2.14), figure 2*b*. This model is similar in character to the models of pancreatic β cell dynamics described in this section; the receptor cell population varies nonlinearly with leptin concentration, meaning that at low concentrations the receptor population increases whereas at higher concentrations leptin receptors die off, increasing food intake. The system can undergo a bifurcation leading to the creation of a stable equilibrium at a high level of adiposity and the destruction of the previous healthy equilibrium.

It may be possible to combine multiscale models with continuous glucose monitoring data to provide estimates of the rate of progression towards diabetes in prediabetic or otherwise high-risk patient groups. By using the continuous glucose monitoring data to estimate insulin levels, and thus pancreatic response to glucose load, measures of insulin sensitivity and β cell function may be tracked over time. It has already been shown that this information can be extracted from intravenous and oral glucose tolerance test data by using models outlined in this section [61]—given the advances in both models and sensor technology since this work was done it is highly likely that it can be adapted to continuous glucose data, leading to more effective screening and preventative action.

## 4. Body composition models have a vital role to play in precision medicine

**Figure 3.**
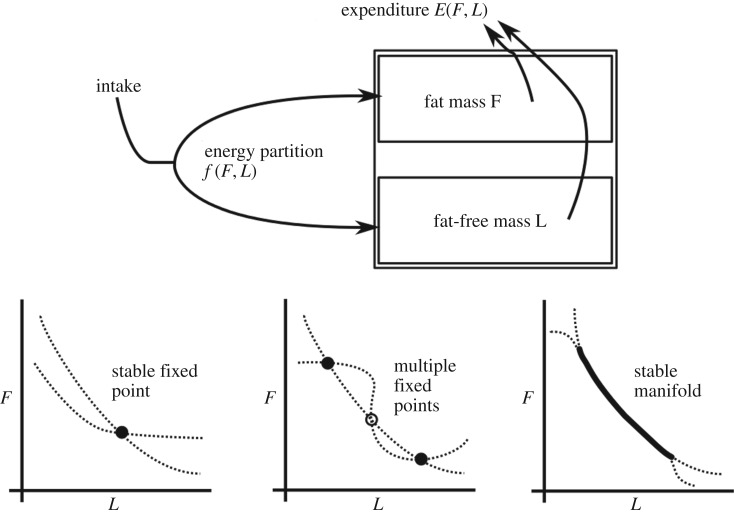
Multiple-compartment models can have different stability properties depending on the rules governing energy partitioning and expenditure. These stability properties can lead to significant differences in physiological outcomes—at a stable fixed point any disturbance, such as a change in energy intake, will lead to compensatory changes that return the system's state to the fixed point. Multiple fixed points are similar, except that the system will reach differing fixed points depending on its state, so potentially large nudges may be needed to move from one fixed point to another. The existence of two stable fixed points implies the existence of an unstable fixed point. Finally, the system is stable at all points along a stable manifold, so small perturbations allow the system to be nudged to other states on the manifold.

Once an animal has eaten, the energy provided by the chemical bonds in the food cannot be destroyed, but must be used by the organism, stored in new chemical bonds, or dissipated as heat. This simple constraint has inspired models which equate the energy flux into an organism from its food with the above expenditures. In these models, the body is typically split into multiple compartments representing different components such as fat, non-fat tissue and circulating reserves (figure 3*a*) and expenditure is taken to depend on energy intake and the composition of these compartments. The dynamics of body composition then depends on the partition of energy between expenditure and storage in adipose tissue. Although our understanding of the physiology of the system is sufficient to specify different components of energy expenditure such as specific dynamic action (i.e. the thermic effect of feeding), basal metabolic rate and expenditure due to physical activity, these models typically make no predictions about energy intake from feeding. The final requirement for specifying such a model is a set of laws characterizing how energy is partitioned among the various compartments. Changes in body composition typically occur over long timescales, so energy partition models focus on long-term dynamics and often do not model short-term behaviour. This can be accomplished in a rigorous mathematical way by a technique known as separation of timescales, in which the short-term behaviour is averaged out and integrated into the long-term system (see box 2); however, this relies on a number of assumptions that may not always be fulfilled. By mathematical analysis of these systems it is possible to determine how they will behave in different circumstances, for instance, if they will tend towards fixed body compositions, or whether a wide range of compositions are possible. In the section below, we review these energy partition models and their properties.

### 4.1. The energy balance model predicts body mass and composition changes over long timescales

A substantial number of energy partition models have been formulated [62–67] (electronic supplementary material, S3.2–S3.7). The model that has been most extensively theoretically developed and experimentally verified has been proposed by Guo & Hall for mice [68,69] and later applied to humans [70]. This model considers energy intake due to carbohydrates, fat and protein, and storage in fat mass, fat-free mass and blood glucose. Over long timescales the system is taken to be in average carbohydrate balance and glucose stores in the blood are neglected, leaving a two-compartment model predicting the dynamics of fat and fat-free mass over weeks and months. The law governing the partitioning of energy between the two compartments is Forbes' law [71,72] (electronic supplementary material, S3.1), which states that the rate of change of fat-free mass with respect to fat mass grows exponentially with increasing adiposity. This quantifies our intuitive understanding that, without significant muscle growth, increases in weight are largely due to increased fat deposition, and that initial body composition has a significant effect on the final state. Forbes’ law has significant empirical justification for adult humans under normal conditions but less so for infants or for adults in situations where body composition changes significantly for reasons other than weight loss (such as when undergoing resistance training) or for other species.

The energy balance model (electronic supplementary material, S3.2) has been verified against both human and mouse data [68–70] and adapted to model the dynamics of body composition in growing children [73]. The energy balance model has also been applied in a public policy setting to evaluate the impact of food wastage in the USA by providing an estimate of the energy requirements of the population, allowing food wastage to be calculated as the difference between estimated food purchases and calorie requirements [74]. If given data on food intake, the energy balance model agrees well with experimental data on body weight and composition, indicating that if it could be combined with a computational model of food intake, the resulting model may be able to accurately predict [62] long-term body composition dynamics.

An alternative approach is to derive results based on how the components of energy homeostasis scale with body size. Kozusko [67] considers a model of this kind with energy expenditure varying as a linear function of body weight (electronic supplementary material, S3.6). Metabolic scaling with body size has been widely investigated in ecology, with a number of scaling relations suggested [75–77]. These scaling relations form the basis of work by Antonetti [66] (electronic supplementary material, S3.7) which considers the body-size scaling of basal and activity-based energy expenditure. This approach has the appealing property of being relatively organism-independent, as some scaling laws have been observed to hold over a wide size range. However, it should be noted that scaling laws have been the subject of some controversy and that inter-species scaling may obey a different law to intra-species scaling [78,79].

### 4.2. Dynamic energy budget theory derives general growth and scaling laws from simple assumptions

Dynamic energy budget (DEB) theory [80–82] (electronic supplementary material, S3.8) is a general theory of growth and maturation which respects stoichiometric constraints, i.e. the conservation of total number of carbon, nitrogen and other molecules. It is not designed with reference to any particular organism, but instead to be able to match any organism through changes of parameters in the model and possibly extensions to the basic theory. In the basic formulation of DEB theory the body is divided into three compartments, in contrast to the two in the basic energy partition model. These compartments are denoted ‘reserve’, ‘maturity’ and ‘structure’, and energy is allocated from intake to each compartment based on a series of partitioning rules. These compartments do not necessarily map directly to individual organs or components of an organism, but rather represent the activities the organism prioritizes expending energy on. Organisms grow by allocating energy to maturity, after which they can then allocate energy to reproduction if energy availability permits, leading to the generation of offspring. Each compartment entails costs both for growth and maintenance, causing energetic costs to increase with growth. The basic DEB theory model allows derivation of a number of well-known results, such as Kleiber's law of metabolic scaling [76] and the growth law of von Bertalanffy [83,84]. DEB theory offers a widely applicable framework for predicting growth and development, while also respecting fundamental stoichiometric constraints.

### 4.3. Many models of energy partition can be reduced to two-compartment models which can be analysed using dynamical systems theory

Energy partition models share a common structure, suggesting that it is possible to analyse the properties of all such systems and identify the key factors that determine their behaviour. Chow & Hall [85] performed such an analysis on two-compartment models, and identified that all such models must possess fixed points, and that the nature of these fixed points (see box 1) will be determined by the functional forms of energy expenditure and the fraction of energy derived from fat (electronic supplementary material, S3.9). Depending on the nature of these two functions, for a given intake there may be a single fixed point, a multitude of discrete fixed points, a continuum or an unstable fixed point with a stable limit cycle around it, as illustrated in figure 2*b* and detailed in box 1. These correspond to very different physiological outcomes. In the first case, the system will always attempt to defend a fixed body composition, and any attempt to alter this will be fighting against the natural dynamics of the body. In the second case, there is more hope—it may be possible to move from a physiologically dangerous fixed point to one which is less dangerous through a perturbation of sufficient size. The third case is even more optimistic—small perturbations may disturb the system's state along the continuum of fixed points, meaning that small, gradual changes are possible. Finally, if a limit cycle exists then weight will naturally oscillate over time through a predetermined pattern to which it will return after any small perturbation. Chow & Hall find that the energy balance model discussed previously possesses a continuum of fixed points if there is no correlation between feeding behaviour over multiple days. It is not clear what effect more complex stochasticity might play on the behaviour of the system, as it has been found to have surprising and complex effects in other dynamical systems analyses, e.g. [86].

### 4.4. Individualizing energy balance models

Inter-individual variation in energy balance has received considerable attention both theoretically and experimentally. Energy intake and expenditure both vary substantially between individuals, with basal metabolic rate [87], dietary induced thermogenesis [88] and absorption of energy from ingested foods [89,90] exhibiting the most variation. Inter-individual variability in basal metabolic rate is particularly important to consider as currently a substantial amount of variation cannot be predicted by known covariates such as body weight and composition [87]. It is possible that hierarchical modelling may resolve this issue in the same way as it has been applied to glucostasis. A further source of variation that has not been considered is the possibility that the partitioning law in the Forbes model may vary between individuals: some may be more predisposed to deposit energy as fat than others. Although a simulator of the energy balance is available online [91], it does not allow these parameters to be estimated from data. Given the explosion of connected consumer devices such as body composition measuring scales, food tracking and exercise logging apps and heart rate enabled activity trackers, the data required for individualized energy expenditure estimation is rapidly becoming available and easy to collect. This personalization of energy balance data might allow for more accurate calibration of required energy intake and expenditure, perhaps leading to more successful weight loss. On its own this will not solve the obesity epidemic; however, energy partition models have been used to compare predicted weight loss under a calorie-restricted diet with the observed weight loss [92]. Even accounting for variation within individuals, these diets have dramatically less effect than they should. A careful model-based study of possible causes identified failure to comply with low-calorie diets as the main reason they fail. Clearly traditional low-calorie diets are hard to maintain. However, it may be that by understanding determinants of eating behaviour and satiety we can construct individualized diet plans that maximize satiety while keeping energy intake low. To do this requires short-term models of feeding behaviour, which we discuss in the next section.

## 5. The importance of stochastic behavioural models for precision health

As we have seen, it is possible to predict the effects of regulatory dysfunction through modelling techniques, and in the near future it may be possible to optimize the treatment of type 1 diabetes by using models to more accurately predict individual blood glucose response to food or insulin administration. These techniques may be applicable beyond type 1 diabetes, however, for example in predicting deviations from planned diets. To do this will require an understanding of behaviour on short timescales, at the resolution of individual meals. This is the scale at which diets fail: although low-calorie diets can produce weight loss initially, their failure to produce sufficient satiety leads to loss of diet adherence in the longer term. The means by which food evokes satiety are complex; however, good proxies for satiety levels are time until the next meal or snack, and the calorie content of this feeding episode. Again, the explosion of data from wearable devices and food logging apps offers new opportunities to collect datasets orders of magnitude larger in both duration and sample size than those used previously in most studies of human feeding behaviour. Leveraging these data alongside pre-existing models and machine learning techniques may allow for personalized diet plans that maximize satiety at a given level of caloric intake. Some plausible candidates for mechanisms by which this could be accomplished include high-protein preloading prior to a meal [41], altered nutrient composition [93] and improved sleep quality [94] among many others. More speculatively, personalized predictive modelling could be used to support behavioural approaches to treating metabolic disease, for example predicting when waning satiety or nadir blood glucose is likely to result in increased hunger, allowing users to ensure they have alternative activities or healthy snacks available to avoid temptation. In this section, we discussed models for regulation of feeding behaviour, emphasize the importance of stochasticity and suggest ways forward for this under-developed area of modelling.

### 5.1. Control-theoretic models have succeeded at long and very short timescales, but meal-level behaviour has been neglected

The ideas of homeostasis and control are closely linked and have been well-studied in mathematics and engineering. Norbert Wiener—a pioneer in the understanding of homeostasis—was also deeply involved in problems of machine control in the presence of uncertainty, particularly through use of feedback mechanisms [95–97]. Feedback control relies on the integration of multiple signals, which are then integrated to yield some behavioural output. Behavioural control differs from most control mechanisms in that control can only be exerted through discrete events such as feeding, rather than in a continuously varying way, for example through a continuous increase in insulin secretion. This makes modelling control of ingestion at the level of individual meals relatively challenging as many of the mathematical techniques of control theory are not applicable. These techniques have some applications at higher or lower time resolutions, such as in the control of feeding rate within a meal or over a period of months. Davis & Levine formulated a model in which feeding input is regulated by a control circuit which incorporates a negative feedback loop reducing intake when the gut fills in a manner similar to a proportional-integral controller (a control mechanism which adjusts the strength of feedback based on the difference from some desired value and the duration for which this difference has existed [98]). This theory obtained good agreement with prior experimental data [99] (electronic supplementary material, S4.1); however, it only models ingestive behaviour in a single feeding bout.

Control-theoretic models are a natural way to investigate the thrifty gene hypothesis. At a longer timescale, more standard differential equation models can again be applied by averaging out feeding behaviour to a continuous arrival of food. A model of leptin-mediated control of feeding behaviour compared the set-point and settling-point hypotheses by using different control architectures [100] (electronic supplementary material, S4.2), showing that neither hypothesis can fully explain energy homeostasis. Set point models fail to recapitulate diet-induced obesity, whereas settling point models fail in low-calorie conditions. A model in which integral control only activates below a threshold achieves better results, with weight gain less tightly controlled than weight loss. This is analogous to the ‘drifty gene’ model proposed by Speakman and colleagues [101,102]. Jacquier *et al.* [103] propose a multisystemic model incorporating ghrelin, glucose and leptin-mediated control of feeding with the energy balance model from the previous section (electronic supplementary material, S4.3). Although the idea of determining feeding behaviour from underlying endocrine data is interesting, glucose and ghrelin levels typically fluctuate largely in response to individual meal bouts, which are averaged out in this model. This makes the interpretation of changes in these endocrine time series unclear, and they would seem to be more naturally included in a short-term feeding model.

To our knowledge the only stochastic model of feeding at the level of individual bouts is a model based on calorie flows formulated by Booth & Toates [104,105] (electronic supplementary material, S4.4). This model incorporates feeding and energy expenditure, which has been tested against experimental data [106]. One of the predictions of this model is that gut filling is the feedback signal driving the multiple small feeding bouts that are observed in mice and rats (rather than, for example, a single long bout). Although this model has had some success, it is only weakly stochastic and so generates trajectories that appear unnaturally regular. This limits both its ability to predict meal timings and to quantify its level of uncertainty about them. It is also quite complex, with many internal variables, and provides no natural way to infer parameters which govern behaviour—these must be set by manual tuning.

Another approach to control problems is based on optimality: given a mathematical description of the dynamics of a system, the constraints on how it can be controlled, and a way of scoring the quality of a given control strategy (this scoring is known as the cost function or fitness) the optimal control can often be derived. This provides the best possible strategy for that cost function. This approach was reviewed by McFarland [107], who, with Sibly & McFarland [108], applied it to a model of animal feeding and drinking (electronic supplementary material, S4.5). A common criticism of optimality arguments is that the choice of cost function can appear arbitrary, but can have a profound impact on the optimal control policy selected. Despite this, in the context of energy homeostasis, energy-balance based cost functions can be a natural choice and have been used to predict nontrivial behaviour in other organisms [109]. Optimality arguments have been useful in the study of other classes of behaviour, for example, work by McNamara & Houston on fitness in relation to reproductive ability at the end of a finite time window, with the specific example of a bird which can choose to forage or perform nonforaging tasks which improve its reproductive chances [110] (electronic supplementary material, S4.6).

### 5.2. Models of learning and reward exist, but have yet to be applied to feeding behaviour

An appealing formalism, and one which incorporates the stochasticity inherent in studying behaviour on a short timescale, is that of Markov Decision Processes. In a Markov Decision Process agents possess a ‘stochastic policy’ which governs how likely they are to pick a course of action given their state. This policy can be well-adapted to the environment if it leads to frequently selecting beneficial choices, where ‘beneficial’ is defined by some reward function analogous to the cost function in optimal control. A model of this type has recently been formulated for working for brain stimulation reward in rats where theory showed good agreement with experimental data [111] (electronic supplementary material, S4.7). Developing models of this type for feeding behaviour presents challenges, however, as brain stimulation can be considered to always provide a constant level of reward whereas the reward provided by feeding is almost certain to depend on an animal's nutritional state.

Finally, a modern approach to understanding behaviour at the neuronal level is through inverse reinforcement learning [112]. In this approach, the system is modelled as a Markov Decision Process with unknown reward function, which is inferred through observing examples of the system's behaviour. Once this reward function has been learnt, the model can then be used to reproduce behaviour similar to that of the system being modelled. This has been successfully applied to thermotactic behaviour in *Caenorhabditis elegans* [113] (electronic supplementary material, S4.8) and is likely to be applicable to other homeostatic behaviours such as feeding. Applying inverse reinforcement learning to neuronal firing data from modern imaging techniques [114] could provide a natural interpretation of the inferred reward function and way to integrate results such as the negative valence of AGRP neuronal activation [115]. Model-derived features such as stomach filling could provide insights into how peripheral signals are integrated in the brain to drive behaviour. However, interpreting models derived from inverse reinforcement learning is challenging and is a current area of research. A possible model-based way to understand neuronal firing data is through neuronal mass models. These are simpler to construct, model, and interpret than stochastic models of individual neurons, and consider neuronal activation at the population level. They have previously been used to understand regulation of the sleep–wake cycle and its response to perturbations [116] (electronic supplementary material, S4.9). Using modern imaging techniques it may be possible to fit neural mass models to population level firing data (for example, [117]) to understand the effect of endocrine drives on feeding.

### 5.3. Stochasticity at the level of meals is a crucial missing link in understanding homeostatic behaviour

**Figure 4.**
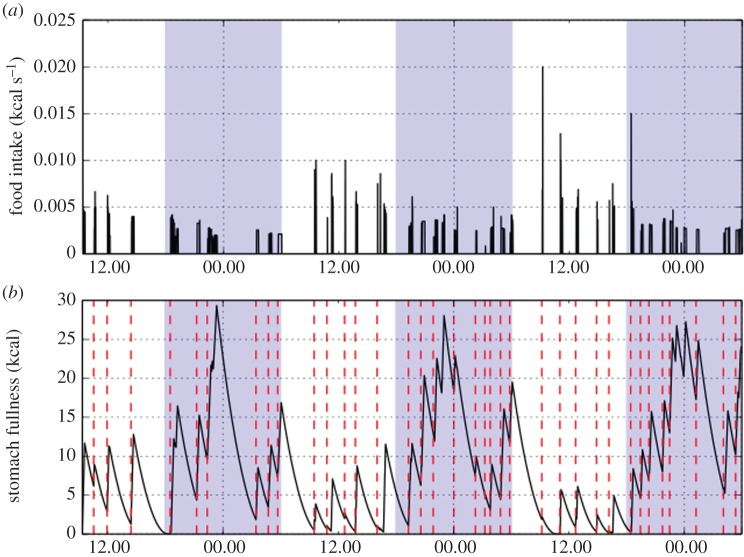
Apparent stochasticity in inter-meal intervals is partially explained by stomach fullness: when the stomach is empty, feeding bouts are very likely to commence. (*a*) Feeding bout data indicating time, duration and average feeding rate. Each meal is composed of multiple feeding bouts, and terminated with a longer pause. Shaded areas indicate dark period (1800–0600). Data are from a male Wistar rat recovering from a fast, observed using an open-circuit comprehensive laboratory animal monitoring system (CLAMS; Columbus Instruments, OH, USA). (*b*) Feeding data are converted to calculated stomach fullness by use of the model for gastric emptying in [104]. Daytime feeding terminates at a lower level than feeding in the dark period (shaded area, as above), and stomach fullness reaches a characteristic peak around midnight.

In general behaviour comes about through the interplay of multiple competing drives—for instance drives for food, water and for sleep. As we have spent the majority of this review showing, most regulatory phenomena are most naturally modelled through continuously varying physiological states, for example glucose/insulin levels and endocrine responses, stomach filling and patterns of body composition. In spite of this, behaviour is definitely not deterministic—rats do not start and stop feeding like clockwork. Nevertheless, we expect that the physiological state of the rat does exert a strong influence on when rats switch between behavioural states. This is backed up by data—when we applied a simple stomach emptying model [104] to experimental data we found a remarkable linkage between stomach fullness and both meal initiation and termination (figure 4). Although the feeding bouts appear random when considered on their own, looking at stomach fullness alongside the feeding data shows an important underlying structure, as well as patterns of day/night variation.

The interrelation between a stochastically switching behavioural state and a continuously varying deterministic physiological state falls between two of the major paradigms of stochastic processes. Markov chains model switching between discrete states; however, these switches typically happen at a constant rate and so fail to capture the dependence on the physiological state. Stochastic differential equations model stochastic changes in continuous variables, but do not offer any way to couple this to a discrete behavioural state.

An appealing alternative way to model homeostatic behaviour is through use of Piecewise Deterministic Markov Processes, also known as stochastic hybrid models [118]. These are generalizations of Markov chains that provide precisely the properties we want: a set of discrete states corresponding to different behaviours, each of which leads to different dynamics on a set of continuous variables corresponding to the animal's physiological state. An application of Piecewise Deterministic Markov Processes to feeding behaviour would be to consider a model with three states: feeding, short pauses within a meal and long pauses which terminate a meal. To capture the behaviour shown in figure 4 we would expect that the length of a long pause be dependent on stomach fullness, and the probability of entering a long pause should grow as the stomach fills. This review has primarily considered continuously varying physiological models; however, the formalism we have outlined here allows for a natural coupling of these mechanistic models to models of behaviour.

A second approach to predicting feeding behaviour is through machine learning tools. As we have seen, these have had some success at predicting quantitative outcomes, for example post-prandial glucose response and blood glucose. The difference here is in the level of predictability of the data. As can be seen in figure 4, although model-derived features (in this case stomach fullness) are informative of feeding behaviour, there is still a substantial amount of variability. It may be that this can be accounted for by enhancing the feature set, for example by including movement and energy expenditure data, however it is possible that behaviour is inherently less predictable than mechanistic responses such as glucostasis, in which case a more detailed understanding of stochasticity may be required, incorporating insights from the large behavioural datasets arising from wearable devices and other personal omics technologies.

## 6. Conclusion

We have brought together diverse areas of modelling in energy homeostasis covering endocrine regulatory systems with a specific emphasis on glucostasis, models of body weight and composition over time, and models of behaviour across multiple timescales. This review has been written to be accessible to the non-mathematician, but we direct the interested reader to our extensive electronic supplementary material where we outline the mathematical details of many of the models we highlight. In each case it has become clear that the advances needed to translate these models into useful tools is individualization. Fortunately, the comprehensive datasets needed to do this are rapidly becoming available through wearable technology and activity trackers. Machine learning techniques offer an appealing way to learn from this large quantity of data, however they can be enhanced by leveraging the decades of physiological understanding represented in the mathematical models reviewed in this article to engineer improved features that can lead to better predictions

The key area for development is in short-term models of feeding behaviour, with resolution of a single meal. By learning from both data and prior experiment how to maximize satiety without increasing calories it may be possible to provide individualized diets that help prevent the failures of compliance typically associated with long-term low-calorie diets. There are technical challenges to overcome, particularly in individualizing physiological models for feature engineering and correctly understanding the type of stochasticity associated with feeding behaviour. If these can be dealt with, the mathematical and machine learning models outlined in this review may prove central to combating the growing obesity epidemic by simply providing, in a dynamic and personalized manner, the right information and guidance for people to make healthier choices.

## Supplementary Material

Mathematical supplement

## Data accessibility

This article has no additional data.
