# Supplementary material for: Quantitative approaches to energy and glucose homeostasis: machine learning and modelling for precision understanding and prediction
Source: J R Soc Interface. 2018 Jan 24;15(138):20170736. doi: 10.1098/rsif.2017.0736 (PMC5805973; doi:10.1098/rsif.2017.0736)
Supplement: Mathematical supplement [file rsif20170736supp1.pdf]

Quantitative approaches to energy and glucose homeostasis: machine learning and modelling for precision understanding and prediction
Supplementary Material S1

Thomas M McGrath[a], Kevin G Murphy[b], Nick S Jones[a]

[a]*Department of Mathematics, Imperial College London*
[b]*Department of Medicine, Imperial College London*

Contents

1 Introduction **2**

2 Endocrine models **2**

2.1 Bergman - Minimal model of insulin secretion . . . 3
2.2 Bolie - Simple insulin and glucose dynamics . . . 3
2.3 Li, Kuang and Mason - Delay differential equation models of glucostasis . . . 4
2.4 Roy and Parker - Extending Bergman's model . . . 4
2.5 Dalla Man - Nonlinear glucose absorption model used in the Type 1 Diabetes simulator . . . 5
2.6 Lehmann and Deutsch - Trapezoidal gastric emptying kinetics . . . 6
2.7 Elashoff model - Modified power exponential gastric emptying . . . 6
2.8 Neural networks - Overview . . . 7
2.9 Time series methods - Overview . . . 7
2.10 Support Vector Regression - Overview . . . 8
2.11 Topp et al. - Multiple-timescale analysis of glucostasis reveals the routes to beta cell extinction . . . 9
2.12 Wang, Khan and van den Berg - Multiscale modelling of glucostasis shows the importance of short-term differences . . . 10
2.13 Sturis and Tolic - Oscillatory insulin delivery increases hypoglycemia . . . 11
2.14 Jacquier et al. - Dynamical systems analysis of a model of leptin resistance and feeding predicts onset of obesity . . . 12

---

*Corresponding author

3 Energy balance models **13**
3.1 Forbes, Hall's updated Forbes' Law - Lean and fat mass partitioning 13
3.2 Hall et al. - Energy balance predicts body composition dynamics 14
3.3 Alpert - Body composition dynamics in hyper- and hypophagia 15
3.4 Dugdale and Payne - Stochastic energy partition model 16
3.5 Westerterp and Speakman - An alternative to set- and settling-point theories 17
3.6 Kozusko - Empirically-derived nonlinear energy partitioning 17
3.7 Antonetti - Energy balance incorporating thermic effect of feeding 18
3.8 DEB - Generalised energy flow model 19
3.9 Chow and Hall - Dynamical systems analysis of the Energy Balance model . 20

4 Control theoretic models **21**
4.1 Davis and Levine - Proportional-integral control of a single feeding bout . . . 21
4.2 Tam et al. - Leptin-mediated control of body weight 22
4.3 Jacquier et al. - Endocrine control of body composition usng the Energy Balance model 23
4.4 Booth et al. - Multiple-factor control of rat feeding 24
4.5 McFarland and Sibly - A general framework for optimal behaviour 25
4.6 McNamara and Houston - Dynamic programming for optimal behaviour . . . 26
4.7 Niyogi et al. - Optimal stochastic policies in working for a reward 27
4.8 Yamaguchi et al. - Learning rewards from behaviour 28
4.9 Fulcher, Phillips, and Robinson - Neural mass theoretic treatment of sleep/wake cycle quantitatively predicts behaviour 29

1. Introduction

In this supplementary document we provide the basic mathematical details of each model detailed in the manuscript. We provide a brief overview of each model, detailing its most important features, before giving the equations characterising the model. We then summarise the main findings of each model, including experimental comparison where appropriate. Further details, such as bifurcation analyses or derivation of optimal controls are not included in full for reasons of brevity, however they are summarised at the end of each section. Readers are directed to the paper in which these details are derived for full details. Although we have altered some notation for ease of reading (especially where variable names were several characters long) we have remained as close as possible to the notation of the original papers, making transitioning into reading the primary literature as simple as possible.

2. Endocrine models

Models in this section describe the dynamics of one or more endocrine regulatory systems in response to external perturbations. The most extensively modelled regulatory system is glucostasis - the regulation of blood glucose by insulin and glycogen. In this case external stimuli are infusions of glucose. This class of models has a surprisingly rich phenomenology,

especially when considered over long timescales and extended to include the dynamics of pancreatic β cells (which secrete insulin, and whose growth depends nonlinearly on glucose concentration). Insights from glucostasis have been used to investigate other mechanisms such as leptin-mediated control of feeding, and models of the interaction between multiple regulatory systems and behaviour are beginning to be investigated.

2.1. Bergman - Minimal model of insulin secretion

Bergman's famous 'minimal model' [1] aims to describe the response of body to the intravenous glucose tolerance test (IVGTT). In the IVGTT glucose is injected as a bolus into the system, and a first-phase insulin secretion is also modeled as a bolus arriving along with the glucose. This model improves on the work of Bolie by considering a second-phase insulin secretion at the pancreas I rather than having a single insulin variable representing the average concentration in the body. This incorporates the fact that it takes time for insulin to travel from the pancreas to the rest of the body, an idea which is developed further in delay differential equation models [SI 2.3]. Changes in I occur in response to dynamics of glucose G and insulin in the 'remote compartment' X, where insulin only acts once it has made its way to the remote compartment. This model leads to a set of 3 ODEs (following the notation of de Gaetano and Arino [2])

$$\frac{dG(t)}{dt} = (P_1 - X)G(t) - P_1 G_b \tag{1}$$

$$\frac{dX(t)}{dt} = -P_2 X(t) + P_3(I(t) - I_b) \tag{2}$$

$$\frac{dI(t)}{dt} = P_4(G(t) - P_5)^{+} t - P_6(I(t) - I_b) \tag{3}$$

The baseline levels of glucose and insulin are given by G_b and I_b respectively, P_1 is the insulin-independent rate of glucose uptake by the body, P_2 the rate of decrease in tissue glucose uptake and P_3 is the insulin-dependent rate. P_4 is the rate of pancreatic release of insulin per mg/dl of glucose, P_5 is the target glycemia in the pancreas and P_6 is the decay rate of insulin in the blood. By fitting time series to this model, a number of metabolic parameters representing glucose tolerance can be obtained, allowing classification of individuals into groups of high and low tolerance.

2.2. Bolie - Simple insulin and glucose dynamics

In 1961, Bolie defined a two-component ODE model of insulin-mediated glucose regulation [3]. In a system of volume V, insulin X and glucose Y are modelled as a system of two coupled ODEs with arbitrary clearance functions F_i

$$V\frac{dX}{dt} = \dot{I} - F_1(X) + F_2(Y) \tag{4}$$

$$V\frac{dY}{dt} = \dot{G} - F_3(X, Y) - F_4(X, Y), \tag{5}$$

where $\dot{I}$ and $\dot{G}$ are rates of arrival of insulin and glucose into the system. Normalising by volume V and linearising the functions F_i around a baseline level $x = X_0 - X$, $y = Y_0 - Y$ with constant insulin and glucose arrival rates p and q gives

$$\frac{dx}{dt} = p - \alpha x + \beta y \tag{6}$$
$$\frac{dy}{dt} = q - \gamma x - \delta y. \tag{7}$$

This system of equations captures rate of insulin secretion as depending on a baseline rate p as well as insulinase with sensitivity α and glucose-dependent pancreatic secretion with rate constant β. Glucose enters the system at rate q, and is stored as glycogen at rate γx and used by tissues at rate δy. This system resembles a damped harmonic oscillator, and can be solved for an initial bolus injection of insulin D_I or glucose D_G. If the coefficients are set so as to prevent oscillations and give a smooth return towards baseline, the dynamics for a glucose tolerance test are

$$y = \frac{D_G}{V}\left(1 - \frac{\delta - \alpha}{2}t\right) e^{-(\delta+\alpha)/2t} \tag{8}$$
$$x = \beta \frac{D_G}{V} t e^{-(\delta+\alpha)/2t}. \tag{9}$$

Bolie also derives dynamics for an insulin tolerance test and general equations where the critical damping criterion is relaxed.

2.3. Li, Kuang and Mason - Delay differential equation models of glucostasis

Li, Kuang and Mason [4] write delay differential equations for glucose G and insulin I to model the delays associated with remote insulin secretion in the pancreas, rather than by incorporating a separate 'remote' compartment. They use the same functions f_1 - f_5 as Sturis and Tolić [SI 2.13], leading to two coupled delay differential equations

$$\frac{dG}{dt} = G_{in} - f_2(G(t)) - f_3(G(t))f_4(I(t)) + f_5(I(t - \tau_2)) \tag{10}$$
$$\frac{dI}{dt} = f_1(G(t - \tau_1)) - d_i I(t). \tag{11}$$

Simulating this system numerically shows the existence of self-sustaining oscillations in glucose and insulin levels. This system is similar to that of Sturis and Tolić, with the hard delay kernels in Li, Kuang and Mason serving the same purpose as the auxilliary variables x_1 - x_3 in Sturis and Tolić. A similar model has also been developed for the IVGTT where the remote compartment is replaced by a delay kernel [5].

2.4. Roy and Parker - Extending Bergman's model

Roy and Parker [6] extend the model of Bergman [SI 2.1] to include free fatty acids (FFAs), which are another source of energy for the body and are particularly heavily used by

skeletal muscle, and infusion protocols other than a simple bolus. Plasma FFA is modeled similarly to glucose in the Bergman minimal model with a remote compartment Z and plasma compartment F. A further variable Y represents the action of insulin to store or utilise FFAs. The ODEs of the model are

$$\frac{dI}{dt} = -nI(t) + p_5 u_1(t) \tag{12}$$
$$\frac{dX}{dt} = -p_2 X(t) + p_3 I(t) \tag{13}$$
$$\frac{dG}{dt} = -p_1 G(t) - p_4 X(t)G(t) + p_6 G(t)Z(t) + p_1 G_b - p_6 G_b Z_b + \frac{u_2(t)}{V_G} \tag{14}$$
$$\frac{dY}{dt} = -p_{F2} Y(t) + p_{F3} I(t) \tag{15}$$
$$\frac{dF}{dt} = -p_7 F(t) - p_8 Y(t)F(t) + p_9(G)F(t)G(t) + p_7 F_b - p_9(G)F_b G_b + \frac{u_3(t)}{V_F} \tag{16}$$
$$\frac{dZ}{dt} = -k_2 Z(t) + k_1 F(t) + k_2 Z_b - k_1 F_b, \tag{17}$$

where p_i are rate constants of the model (see [6] for details), apart from p_9 which is a function of G. Variables with subscript b (G_b, Z_b, F_b) are baseline values of the respective time-varying concentrations and V_G, V_F are the volumes in which glucose and free fatty acid are distributed. Endocrine concentrations vary in response to exogenous stimulation: $u_1(t)$ is the exogenous insulin infusion, $u_2(t)$ is the arrival rate of glucose and $u_3(t)$ is the arrival rate of lipid. Roy and Parker fit the model to experimental data and find $R^2 \geq 0.8756$ after the fitting procedure. High fatty acid concentrations impair glucose uptake in both the model and experiments. The model further predicts that insulin will have an antilipolytic effect, which was verified using experimental data. The addition of the extra compartments was justified by using the Akaike Information Criterion [7], with the improved fit outweighing the penalty from increased model complexity.

2.5. Dalla Man - Nonlinear glucose absorption model used in the Type 1 Diabetes simulator

Dalla Man et al [8] model the stomach as a three compartment ODE, which attempts to reproduce the biphasic nature of gastric emptying. Movement between the stomach compartments is linear, whereas gastric emptying contains a nonlinear term. The variables q_{sto1} and q_{sto2} model the amount of glucose in compartments 1 and 2 of the stomach respectively, q_{gut} is the amount of glucose in the gut and Ra is the rate of glucose absorption into the bloodstream. The system of equations for a given glucose dose D are

$$\dot{q}_{sto1}(t) = -k_{21} q_{sto1} + D\delta(t) \tag{18}$$
$$\dot{q}_{sto2}(t) = -k_{empt}(q_{sto1}, q_{sto2}) q_{sto2} + k_{21} q_{sto1} \tag{19}$$
$$\dot{q}_{gut}(t) = -k_{abs} q_{gut} + k_{empt}(q_{sto1}, q_{sto2}) q_{sto2} \tag{20}$$
$$Ra(t) = f k_{abs} q_{gut} \tag{21}$$

where k_{21} is the rate of movement from compartment 1 to compartment 2, $\delta(t)$ is a delta function, k_{abs} is the absorption rate and f is the fraction of intestinal absorption that reaches the blood. The stomach emptying rate k_{empt} varies with stomach contents q_{sto} according to the equation

$$k_{empt}(q_{sto}) = k_{min} + \frac{k_{max} - k_{min}}{2} \Big(\tanh(\alpha(q_{sto} - bD)) - tanh(\beta(q_{sto} - cD)) + 2\Big) \qquad (22)$$

where the stomach contents q_{sto} is the average of q_{sto1} and q_{sto2}, i.e. $q_{sto} = q_{sto1} + q_{sto2}$. The dual sigmoid nature of the stomach emptying rate causes the stomach to empty slower. A simplified model without this nonlinear emptying rate failed to accurately reproduce the data.

2.6. Lehmann and Deutsch - Trapezoidal gastric emptying kinetics

Lehmann and Deutsch [9] formulated a model of gastric emptying with a single gut compartment q_{gut}. The gut compartment dynamics and glucose arrival rate to bloodstream $Ra(t)$ are given by

$$\dot{q}_{gut}(t) = -k_{abs} q_{gut}(t) + G_{empt}(t) \qquad (23)$$

$$Ra(t) = f k_{abs} q_{gut}(t) \qquad (24)$$

and the fraction of glucose arrival f is calibrated to each individual using the total glucose dose D. This model posits trapezoidal emptying dynamics for $G_{empt}(t)$:

$$G_{empt}(t) = \begin{cases} \frac{tV_{max}}{T_{up}} & t < T_{up}, \\ V_{max} & T_{up} \le t < T_{up} + T_{max}, \\ V_{max} - \frac{V_{max}}{T_{down}}(t - T_{up} - T_{max}) & T_{up} + T_{max} \le t < T_{up} + T_{max} + T_{down}, \\ 0 & \text{otherwise} \end{cases} \qquad (25)$$

where T_{up} is the length of time spent in the rising part of the trapezoid, T_{down} the length of the falling period and T_{max} the time spent at maximum emptying speed. V_{max} is the rate of emptying needed to process all the ingested glucose in the assigned time. Although the kinetics are in principle relatively simple, this trapezoidal structure leads to a relatively large number of parameters.

2.7. Elashoff model - Modified power exponential gastric emptying

The gut dynamics used by Elashoff [10] are identical to the Lehmann and Deutsch model, apart from the stomach contents which is modelled as a power exponential

$$q_{duo}(t) = D(1 - e^{-kt^{\beta}}) \qquad (26)$$

where k is emptying rate and β controls the lag phase of gastric emptying. The rate of gastric emptying is the time derivative of q_{duo}, i.e. $G_{empt}(t) = \dot{q}_{duo}(t)$. This is similar to the model of stomach fullness used by Siegel et al. [11] where the fraction of stomach fullness is given by $q_{frac} = 1 - (1 - e^{-kt})^{\beta}$. Although this is a worse fit than the Dalla Man model, it is substantially more parsimonious with only 2 free parameters.

2.8. *Neural networks - Overview*

Neural networks aim to approximate some nonlinear function $f^*(x)$ with input x. Typically x is a large vector, for instance a series of blood glucose and insulin measurements. Deep networks calculate f through the composition of functions $f(x) = f_1(f_2(x))$. These functions (called activation functions) are parametrised by weights (denoted W) and constants c, which we want to learn from data. For instance, the commonly used rectified linear unit uses the activation function

$$f(x, W, c) = \max\{0, W^T x + c\}. \tag{27}$$

Although simple composition of these nonlinear functions may seem restrictive, with a sufficiently large network it is possible to approximate arbitrary functions. Application of this operation across two layers with no restriction on the weights W_i or constants c_i at layer i gives the network function as

$$f(x, W_1, W_2, c_1, c_2 = f_2(f_1(x, W_1, c_1), W_2, c_2) \tag{28}$$
$$= \max\{0, W_2^T \max\{0, W_1^T x + c_1\} + c_2\}. \tag{29}$$

This yields a vector which can be converted into a scalar prediction (for instance blood glucose at some future time point) by another layer. Layers which are not generating a prediction are referred to as hidden layers. Recurrent neural networks are networks whose structure is optimised for prediction of sequential data. Recurrent neural networks receive inputs at a series of time points x_t, which are used to compute output observations o_t based on hidden units h_t. Hidden units are updated at each time step, i.e. at time t, the hidden units h_t depend only on h_{t-1} and the observations x_t, with loss being calculated from output o_t and data y_t. This encoding of sequential structure makes them ideal for time series prediction tasks, as well as natural language processing. Neural networks are an extremely active area of current research, and an excellent introductory textbook has recently been published [12].

2.9. *Time series methods - Overview*

Time series analysis attempts to separate stochastic and deterministic parts of the dynamics under regulation and learn the parameters of the deterministic part. In general, time series models assume discretised timesteps, where the value of each timestep Y_t is a function of the previous values and some noise ϵ_t:

$$Y_t = F(y_{t-1}, y_{t-2}, \ldots) + \epsilon_t. \tag{30}$$

Given the very broad specification above, there are two possible approaches: attempt to learn a nonlinear function using a range of function approximation techniques (for instance neural networks), or to assume a more tractable model structure and learn parameters of that model directly. We discuss the latter approach here, as it has been widely used in applications to glucostasis. One common tractable model class is linear processes, where the

value at each timestep is a linear function of the preceding ones. Moving average models ($MA(q)$) are linear functions of the noise at the previous q timesteps:

$$Y_t = e_t - \theta_1 e_{t-1} - \theta_2 e_{t-2} - \cdots - \theta_q e_{t-q} \tag{31}$$

so this is a weighted average of the last q noise observations. An autoregressive model ($AR(p)$) uses a number p of past observations

$$Y_t = \phi_1 Y_{t-1} + \phi_2 Y_{t-2} + \cdots + \phi_p Y_{t-p} + e_t \tag{32}$$

where we want to learn the terms ϕ_i. This can be rewritten in terms of e_i as a general linear process. These two are combined to form the autoregressive moving average (ARMA) model. The other important component is the addition of exogenous variables - in this case Y_t is also a function of other variables X_t whose dynamics is unknown and we are simply given. This could be energy expenditure or food intake, for instance. As with support vector machines we do not cover inferring the parameters θ and ϕ from observed time series, however extensive resources are available [13, 14, 15].

2.10. Support Vector Regression - Overview

Support vector machines (SVMs) are a computationally-efficient method for prediction that allow the prediction to be a nonlinear function of the data. The basic idea of an SVM is that although a linear function of the data may not yield accurate predictions, it may be possible to transform the data into some new space where a linear function of the transformed data is a good predictor. SVMs are often used for classification, where the function to be learned is the boundaries between different classes, however we will discuss their application to regression problems [16]. We follow the introductory material given in a comprehensive tutorial [17], but do not cover the optimisation methods necessary to actually learn parameters in SVMs. Begin by attempting to learn a linear predictor f using data x and parameters w, b:

$$f(x) = \langle w, x \rangle + b \tag{33}$$

where $\langle \cdot, \cdot \rangle$ denotes the inner product in the data space (typically $\mathbb{R}^n$, although other inner product spaces are possible). The problem is typically phrased in terms of convex optimisation, with the goal being to minimise the norm of the weights ($\|w\|^2 = \langle w, w \rangle$ such that the regression line never has more than ϵ error with respect to the data y, leading to the constraints

$$y_i - \langle w, x_i \rangle - b \leq \epsilon \tag{34}$$

$$\langle w, x_i \rangle + b - y_i \leq \epsilon. \tag{35}$$

This differs from conventional linear regression, where the aim is to minimise the sum of squared errors and (unless regularisation is being used) there is no constrain on w. This optimisation problem can be unsolvable for some values of ϵ as the data may simply be broadly distributed. To deal with data outside of the margin ϵ, we introduce variables ξ_i

and ξ^* that penalise the distance of each data point from the 'allowed region' within ϵ of the regression line. This leads to the optimisation problem:

$$\text{minimise} \quad \frac{1}{2}\|w\|^2 + \frac{C}{2}\sum_i(\xi_i + \xi_i^*) \tag{36}$$

subject to the constraints

$$y_i - \langle w, x_i \rangle - b \leq \epsilon + \xi_i \tag{37}$$
$$\langle w, x_i \rangle + b - y_i \leq \epsilon + \xi_i^*. \tag{38}$$

This introduces a tradeoff between weight minimisation and error tolerance, which is controlled by the parameter C. Because this formulation allows the tools of convex optimisation to be used, the problem can be solved efficiently. However, if we have nonlinear patterns in the data, we will fail to learn them with this model. We would like transform the data $x \to \Phi(x)$ such that it is linearly separable in the transformed space, however for complex and high-dimensional data (e.g. images) this is computationally infeasible. The problem is resolved using the 'kernel trick'. This relies on the insight that we only use the data via its dot product, never directly. Functions that satisfy a series of properties (stated in [17]) allow us to compute the dot product in the transformed space without needing to calculate the coordinate transforms explicitly. This renders the problem tractable, replacing the inner product $\langle \cdot, \cdot \rangle$ with the kernel $k(\cdot, \cdot)$ in the optimisation equations above. The effectiveness of SVMs depends strongly on the kernel used, although a range of kernels exist, including for more complex data structures such as graphs.

2.11. Topp et al. - Multiple-timescale analysis of glucostasis reveals the routes to beta cell extinction

Topp et al. [18] formulate a multiple-timescale model incorporating fast-timescale regulation of glucose G by insulin I and slow dynamics of pancreatic beta cell mass β. Glucose is described by the following ODE

$$\frac{dG}{dt} = R_0 - (E_{G0} + S_I I)G \tag{39}$$

the rate of production at $G = 0$ is R_0, E_{G0} is the effectiveness of glucose at $I = 0$, and S_I is insulin sensitivity. Insulin clearance rate is taken to be linear in insulin concentration with rate k, whereas production is linear in β and sigmoidal in G with a maximal rate σ:

$$\frac{dI}{dt} = \frac{\beta\sigma G^2}{\alpha + G^2} - kI. \tag{40}$$

The glucose-insulin model evolves on a much faster timescale than the beta cell dynamics, which are given by the balance of replication and death, with replication $R = (r_{1r}G - r_{2r}G^2)\beta$ and death $D = (d_0 - r_{1a}G + r_{2a}G^2))$, giving

$$\frac{d\beta}{dt} = (-d_0 + r_1 G + r_2 G^2)\beta. \tag{41}$$

The baseline beta cell death rate is d_0, and $r_1 = r_{1r} + r_{1a}$ and $r_2 = r_{2r} + r_{2a}$ are the net rate constants for glucose-dependent beta cell growth or death. The system has two stable steady states, one corresponding to a normal, non-pathological state and another at a pathological state where glucose concentration is elevated and beta cell mass diminshes to zero. These are separated by a saddle point. Analysis of this system predicts a number of possible pathways into diabetes: movement of the non-pathological fixed point to a hyperglycemic state, bifurcation eliminating the saddle point and non-pathological fixed point, and an interaction between fast and slow subsytems driving glucose levels up faster than beta cell mass adaptation can cope with.

2.12. Wang, Khan and van den Berg - Multiscale modelling of glucostasis shows the importance of short-term differences

Building on both of the preceding ideas, Wang et al.develop a multiscale model with oscillatory glucose input [19]. They extend Sturis and Tolić's model [SI 2.13] by adding a time-varying glucose source $\psi_{in}(t)$, leading to the following inhomogeneous system of ODEs

$$\frac{dG}{dt} = \psi_{in}(t) - \psi_{II}(G) - \psi_{ID}(G, I_i) + \psi_{GR}(G, w_3) - \psi_{GX}(G) \tag{42}$$

$$\frac{dI_p}{dt} = Q\psi_{IS}(G) - \phi\left(\frac{I_p}{V_p} - \frac{I_i}{V_i}\right) - \frac{I_p}{\tau_p} \tag{43}$$

$$\frac{dI_i}{dt} = \phi\left(\frac{I_p}{V_p} - \frac{I_i}{V_i}\right) - \frac{I_i}{\tau_i} \tag{44}$$

$$\frac{dw_1}{dt} = \frac{3(I_p - w_1)}{\tau_d} \tag{45}$$

$$\frac{dw_2}{dt} = \frac{3(w_1 - w_2)}{\tau_d} \tag{46}$$

$$\frac{dw_3}{dt} = \frac{3(w_2 - w_3)}{\tau_d} \tag{47}$$

$$\frac{dQ}{dt} = (\rho(I_p) - \mu(G))Q(t) + \psi_{NG}(G, I_i), \tag{48}$$

where ψ_{II} is insulin-independent uptake, ψ_{ID} is insulin-dependent, ψ_{GR} is glucose release from hepatic glycogen stores, and ψ_{GX} is glucose excretion. The parameter ϕ is a rate constant for the exchange of insulin between the blood and interstitial spaces, which have volumes V_p and V_i respectively. The mean life time of insulin is τ_i in interstitial space and τ_p in the blood. The delay constant τ_d governs how long it takes for glucose to be released from hepatic glycogen. The differences between this model and the model of Topp et al. are time-varying glucose influx, the addition of insulin dependence in pancreatic beta cell dynamics, and a glucose and insulin-dependent pancreatic beta cell neogenesis term ψ_{NG}. Wang et al. separate the dynamics into slow and fast systems, and analyse the behaviour of the system. They find that β cell mass can be significantly altered by the way that glucose is delivered - the same amount of glucose can lead to much worse consequences if delivered

in several short bursts than if it were continuously infused, showing that short-timescale dynamics can have an effect on long-term onset of pathology.

2.13. *Sturis and Tolic - Oscillatory insulin delivery increases hypoglycemia*

This is an extension of Sturis' earlier work [20] and models intercellular insulin I_i, plasma insulin I_p and glucose G with a gamma-kernel delay of $n = 3$ between plasma insulin and its effect on glucose production given a constant rate of glucose infusion G_{in} [21]. This model is specified by the set of equations

$$\frac{dG}{dt} = G_{in} - f_2(G) - f_3(G)f_4(I_i) + f_5(x_3) \tag{49}$$

$$\frac{dI_p}{dt} = f_1(G) - E\left(\frac{I_p}{V_p} - \frac{I_i}{V_i}\right) - \frac{I_p}{t_p} \tag{50}$$

$$\frac{dI_i}{dt} = E\left(\frac{I_p}{V_p} - \frac{I_i}{V_i}\right) - \frac{I_i}{t_i} \tag{51}$$

$$\frac{dx_1}{dt} = \frac{3}{t_d}(I_p - x_1) \tag{52}$$

$$\frac{dx_2}{dt} = \frac{3}{t_d}(x_1 - x_2) \tag{53}$$

$$\frac{dx_3}{dt} = \frac{3}{t_d}(I_2 - x_3), \tag{54}$$

where E is the transfer rate between plasma and intercellular space, V_i and V_p are the volumes of the interstitial space and plasma respectively and t_i, t_p, and t_d are rate constants. Insulin production in the pancreas is determined by glucose concentration according to the following sigmoidal function

$$f_1(G) = \frac{R_m}{1 + \exp((C_1 - G/V_a)/a_1} \tag{55}$$

where R_m is the maximal release rate and C_1 and a_1 are constants fitted from previous work [22, 23]. The function $f_2(G)$ models insulin-independent glucose utilisation by cells

$$f_2(G) = U_b(1 - \exp(-G/(C_2V_g))) \tag{56}$$

where U_b is the baseline uptake rate and C_2 is a constant which is fitted to prior experimental data [24]. Brain and nerve cells consume glucose in an insulin-independent manner, whereas the consumption of muscle and fat is determined by both glucose and insulin availability through the following equations

$$f_3(G) = \frac{G}{C_3V_g} \tag{57}$$

$$f_4(I_i) = U_0 + \frac{U_m - U_0}{1 + \exp(-\beta\ln(I_i/C_4(1/V_i + 1/Et_i)))}, \tag{58}$$

where U_0 and U_m are rate constants and C_4 is fitted to data. Finally, insulin affects hepatic glucose production according to the following equation:

$$f_5(x_3) = \frac{R_g}{1 + \exp(\alpha(x_3/V_p - C_5)}, \tag{59}$$

where R_g is the maxiumum rate. Tolić et al.simplify their model by replacing the functions f_i with polynomial approximations and analyse its properties. They find that oscillatory insulin delivery has a greater hypoglycemic effect than continuous delivery.

2.14. Jacquier et al. - Dynamical systems analysis of a model of leptin resistance and feeding predicts onset of obesity

Jacquier et al.couple the energy partition model by Hall et al.[SI 3.2] to a model of leptin-mediated food intake as well as a nonlinear model of leptin receptor density in an attempt to model progressive leptin resistance. Energy intake I and expenditure E determine the dynamics of fat mass F and fat-free mass M (with energy densities ρ_F, ρ_M respectively

$$\frac{dF}{dt} = \frac{I - E}{\rho_F \Omega + \rho_M} \tag{60}$$

$$\frac{dM}{dt} = \frac{\Omega(I - E)}{\rho_F \Omega + \rho_M}, \tag{61}$$

where the function Ω is given by Forbes' law. Leptin production rate is modelled as being linearly dependent on fat mass with rate constant γ_L and having degradation rate δ_L

$$\frac{dL}{dt} = \gamma_L F - \delta_L L. \tag{62}$$

The rate of change of leptin receptor density R is assumed to vary quadratically in L

$$\frac{dR}{dt} = \gamma_R(1 + \lambda_{R1} L) - \delta_R(1 + \lambda_{R2} L^2)R, \tag{63}$$

where γ_R is the basal production rate, λ_1 the leptin-dependent growth rate, δ_R the basal death rate and λ_{R2} the leptin-dependent degradation rate. Finally, food intake is given as a saturable function of leptin concentration

$$\frac{dI}{dt} = \frac{\gamma_I}{1 + \Phi_R(L)} - \delta_I I, \tag{64}$$

where the function $\Phi_R(L)$ models the activation of leptin receptors, and is given by a Hill function

$$\Phi_R(L) = \frac{\phi R L^n}{L^n + \theta^n}. \tag{65}$$

A bifurcation analysis shows the existence of stable solutions; as γ_I and λ_{R2} are varied these move from possessing a single healthy solution through to a bistable state where obese or healthy steady states are possible, to a single stable equilibrium in an obese state. Oscillating food inputs can lead to the onset of leptin resistance and subsequent obesity. Comparison of the model to experiments where leptin was continuously injected show a good agreement to the data.

3. Energy balance models

Energy partition models are typically deterministic ODE models which consist of a number of body compartments (such as fat and lean tissue) and a set of functions for partitioning energy intake between them. They aim to forecast body composition changes over time given data for calorie intake and expenditure, but generally make no attempt to predict behaviour.

3.1. Forbes, Hall's updated Forbes' Law - Lean and fat mass partitioning

From a regression study of women across a wide range of ages and levels of adiposity, Forbes found the following regression relationship between lean body mass L and fat mass F [25]

$$L = 10.4 \ln F + 14.2, \tag{66}$$

thus

$$\frac{dL}{dF} = \frac{10.4}{F}. \tag{67}$$

Under this model changes of body composition are taken to lie along the regression line described above - this does not account for changes in body composition due to weight training, for example, however it appears to provide a good model for weight loss or gain in typical females from dietary causes. Assuming that the body is comprised of two compartments L and F such that total body weight $W = L + F$, an infinitesimal change in body weight will lead to a fat-dependent infinitesimal change in lean body mass according to the equation

$$\frac{dL}{dW} = \frac{10.4}{10.4 + F}. \tag{68}$$

Hall generalised Forbes' law to macroscopic changes in bodyweight from an initial fat mass F_i to a final fat mass F_f [26] by using the fact that $\Delta W = \Delta F + \Delta L$ to write

$$\frac{\Delta L}{\Delta W} = \frac{\Delta W - \Delta F}{\Delta W} \tag{69}$$

$$= 1 - \frac{F_f - F_i}{\Delta W}. \tag{70}$$

By expressing ΔL as $L_f - L_i$ and using Equation 66 this can be rewritten as

$$F_f = \exp\left(\frac{L_f - 14.2}{10.4}\right). \tag{71}$$

After some algebra, the macroscopic change in lean mass for a given change in body weight is given by an expression involving the Lambert W function $\mathcal{W}$:

$$\frac{\Delta L}{\Delta W} = 1 + \frac{F_i}{\Delta W} - \frac{10.4}{\Delta W}\mathcal{W}\left(\frac{1}{10.4}\exp\left(\frac{\Delta W}{10.4}\right)F_i \exp\left(\frac{F_i}{10.4}\right)\right). \tag{72}$$

Forbes' law thus predicts that lean mass will increase with increasing body weight, but that higher adiposity will diminish the rate of increase. Thus energy will be partitioned to lean or fat tissue differently depending on the current level of adiposity, contrary to the model of Dugdale and Payne [SI 3.4] in which a constant ratio is assumed (called the P-ratio).

3.2. Hall et al. - Energy balance predicts body composition dynamics

Hall and coauthors have published a number of papers on the subject of energy homeostasis, including predicting dynamics of body weight, composition and fuel selection in humans [27, 28, 29] and mice [30, 31]. These models rely on the principle of energy conservation, and split the organism's body into two compartments - the fat and lean compartments. Energy balance can be specified in terms of energy balance to and from each of these compartments

$$\rho_F \frac{dF}{dt} + \rho_L \frac{dL}{dt} = I - E, \tag{73}$$

where ρ_F and ρ_L denote energy densities of fat and lean tissues respectively, and E and I are energy expenditure and intake. Applying Forbes' relationship and defining $\alpha = F/10.4$ gives

$$\rho_L \frac{dL}{dt} = \left(\frac{\alpha \rho_L}{\alpha \rho_L + \rho_F} \right) (I - E) \tag{74}$$

$$\rho_F \frac{dF}{dt} = \left(\frac{\rho_F}{\alpha \rho_L + \rho_F} \right) (I - E). \tag{75}$$

Accounting for all sources of energy expenditure provides a model for energy expenditure

$$E = K + \beta \Delta I + (\gamma_L + \lambda)L + (\gamma_F + \lambda)F + \eta_F \frac{dF}{dt} + \eta_L \frac{dL}{dt}, \tag{76}$$

where K is the cost of thermogenesis, β a factor to account for the thermic effect of feeding, γ_F and γ_L the metabolic costs of fat and leat tissue and λ the cost of physical activity, which is assumed to vary with body weight. The parameters η_F and η_L are the biochemical efficiencies of synthesising fat and lean tissue respectively; K and λ are determined from model calibration, but β, γ_L, γ_F, η_F and η_L are fixed from previous experiments. Physical activity is taken to be partly determined by diet; in order to determine this relationship, mice were switched to a different diet at timepoints t_1 and switched back at t_2. This data was used to calibrate λ for a variety of diets. Fuel selection is another important quantity predicted by this model - meals with different macronutrient compositions but the same amount of calories may have different effects. Accounting for fat, protein, and glucose intakes separately as I_F, I_P and I_G respectively, and assuming nutrients are either immediately oxidated (O_F, O_P, and O_G respectively), used in de-novo lipogenesis (DNL) or gluconeogenesis (GNG) gives the following set of ODEs

$$\rho_F \frac{dF}{dt} = I_F + DNL - O_F \tag{77}$$

$$\rho_P \frac{dP}{dt} = I_P - GNG - O_P \tag{78}$$

$$\rho_G \frac{dG}{dt} = I_G + GNG - DNL - O_G. \tag{79}$$

It can be shown [30] that these lead to equations for net oxidation of each macronutrient group (where net oxidation is defined as oxidation without de novo lipogenesis or gluconeogenesis)

$$O_{F,net} = I_F - \left(\frac{\rho_F}{\alpha\rho_L + \rho_F}\right)(I - E) \tag{80}$$

$$O_{P,net} = I_P - \left(\frac{\alpha\rho_L}{\alpha\rho_L + \rho_F}\right)(I - E) \tag{81}$$

$$O_{C,net} = I_C. \tag{82}$$

However, these quantities cannot be measured directly in experimental settings. A measurement which can be calculated from gas exchange data is the respiratory quotient RQ, which uses the fact that fat and carbohydrate oxidation consume oxygen and produce carbon dioxide in different proportions to determine the balance of energy production from these sources. Writing RQ in terms of macronutrient consumption gives

$$RQ = \frac{0.7 \times O_{F,net} + 0.83 \times O_{P,net} + O_{C,net}}{E}. \tag{83}$$

Comparing this with the food quotient FQ, which measures the oxygen consumption and carbon dioxide production from combusting the food outside of the body provides a measure of macronutrient imbalance, which can be used to predict body weight and composition changes

$$FQ = \frac{0.7 \times I_F + 0.83 \times I_P + I_C}{I}. \tag{84}$$

Once fitted to experimental data, this theory appears to have widespread applicability in predicting body weight and composition changes for several organisms and in a range of circumstances (see above).

3.3. Alpert - Body composition dynamics in hyper- and hypophagia

Alpert considers a two compartment energy partition model with fat store f and fat free mass l of energy densities α and β respectively [32]. Food is ingested at a rate $\dot{P}$ of which ϵ is digested gives rise to an energy balance equation

$$\alpha\frac{df}{dt} + \beta\frac{dl}{dt} = \epsilon\dot{P} - RMR(l, g) - \delta(1 + f), \tag{85}$$

where energy is lost due to resting metabolic rate RMR, which depends on lean mass l and growth g, and total energy expenditure with activity coefficient δ. The energy available is a fraction ϵ of the total intake $\dot{P}$. A number of further assumptions, including constant or slowly-varying partitioning of excess energy between fat and fat-free mass (denoted by x), lead to a pair of ODEs for body composition in hyperphagia

$$\alpha\frac{d\Delta f}{dt} + \delta\Delta f = x\epsilon\Delta\dot{P} \tag{86}$$

$$\beta\frac{d\Delta l}{dt} + (\gamma + \delta)\Delta l = (1 - x)\epsilon\Delta\dot{P}, \tag{87}$$

with Δ denoting changes from initial value (e.g. $\dot{P} = \dot{P}_0 + \Delta\dot{P}$). These have solutions

$$\Delta f = \frac{x\epsilon\Delta\dot{P}}{\delta}(1 - e^{-\delta t/\alpha}) \tag{88}$$

$$\Delta l = \frac{(1-x)\epsilon\Delta\dot{P}}{\gamma+\delta}(1 - e^{-(\gamma+\delta)t/\beta}). \tag{89}$$

Fitting to experimental data gives $x = 0.87 \pm 0.06$. The model makes a number of surprising predictions: during hyperphagia fat increase is initially independent of activity levels, increasing amounts of energy are partitioned towards fat-free mass, and the fat store is asymptotically bounded. Although Alpert cites some experimental evidence for these claims, the evidence given is not conclusive and seems at odds with an intuitive understanding of body composition during hyperphagia.

3.4. Dugdale and Payne - Stochastic energy partition model

Dugdale and Payne define a computational energy partition model with stochastic energy intake and expenditure [33, 34]. The model is discretised at a one day resolution. Expenditure E and intake I are normally distributed with means μ_E and μ_I and standard deviations σ_E, σ_I respectively. The compartments are tissue fat T, structural fat S, 'fast' lean tissue F and 'slow' lean tissue L, and these are used to derive the energy required for tissue maintenance M according to their masses

$$M = 1.5(\rho_T T + \rho_S S + \rho_F F + \rho_L L), \tag{90}$$

where ρ is the energy consumption per kg of a given compartment. The prefactor is given from a WHO Technical Report estimating minimum energy requirements. This gives the daily calorie balance B as

$$B = I - E - M. \tag{91}$$

Each individual is characterised by the fraction of excess energy deposited to as lean tissue P, with the remainder being deposited as fat tissue. Energy storage is assumed to be inefficient, with only proportions E_1 and E_2 of energy allocated to lean tissue and fat creation respectively actually being deposited. For a day with an energy surplus, tissue deposits are updated by the following rules:

$$\Delta F = QBP/4680 \tag{92}$$

$$\Delta S = 0.08QBP/4680, \tag{93}$$

where $Q = 1 + E_1 P + E_2(1-P)$ and the factor of 4680 converts between energy and body mass. For negative balance the situation is similar, but energy retrieval is taken to be completely efficient and storage fat can also be metabolised

$$\Delta F = BP/4680 \tag{94}$$

$$\Delta S = 0.08BP/4680 \tag{95}$$

$$\Delta T = (1-P)/29390 - 0.08P/4680. \tag{96}$$

Finally, the transfers between fast and slow lean tissue are calculated according to the change in ratio between fast and slow lean tissues. For initial values L_0 and F_0, $K_1 = F_0/L_0$ and $K_2 = F/L$. Now

$$\Delta F = K_1 - K_2 \tag{97}$$
$$\delta L = -(K_1 - K_2). \tag{98}$$

Dugdale and Payne compare their model to a number of underfeeding and overfeeding experiments and find reasonable quantitative agreement, especially in the case of underfeeding obese subjects.

3.5. Westerterp and Speakman - An alternative to set- and settling-point theories

In addition to set- and settling-point theories, Speakman has put forward a model known as the dual intervention point model [35, 36]. In set-point and settling-point models, control of energy homeostasis in scenarios of energy excess and energy deprivation occurs via the same mechanism. In the dual intervention point model, energy homeostasis is weakly regulated between an upper and lower limit, where settling-point behaviour occurs due to environmental factors, and strongly regulated above or below these limits, where set-point control acts on energy homeostasis. This has the advantage of explaining the asymmetry of body weight drift, as the lower limit is set by physical and physiological concerns, and cannot go lower without endangering the organism. In prehistory, the upper weight limit is considered to be irrelevant as in practice high body weights would be selected against by food shortages and predation. However, once predatory pressure and food shortages became less of a consideration, regulation of unhealthily high body weight was left in the hands of physiological mechanisms of bodyweight regulation, which are expected to vary considerably and drift between generations.

A mathematical model of this theory has yet to be developed, although Speakman & Westerterp have formulated a model of weight loss under total starvation that captures some aspects of the theory [37]. It has some similarities to the combination model of Tam et al.[SI 4.2]; if the mechanism of energy sensing is endocrine then formulating a model in terms of two endocrine regulatory systems, one acting on each control threshold, would give this model physiological relevance and test theories of endocrine control. It may also account for the disparity of timescales in endocrine regulation - going below the lower threshold is an immediate threat to life, whereas going above the upper threshold is less optimal, but not immediately dangerous.

3.6. Kozusko - Empirically-derived nonlinear energy partitioning

Kozusko [38] attempts to account for the observed decrease in energy expenditure per unit of body weight by allowing energy expenditure E to depend on body weight W according to the following equation

$$E = \alpha(W)W, \tag{99}$$

where α accounts for the aforementioned variation. In Kozusko's model, α is linear in W away from a setpoint α_0 and body weight W_0 to starvation levels α_s, W_s

$$\alpha(W) = \left(\frac{\alpha_s W_0 - \alpha_0 W_s}{W_0 - W_s}\right) + \left(\frac{\alpha_0 - \alpha_s}{W_0 - W_s}\right) W, \tag{100}$$

which can be rewritten as $\alpha = \alpha_0 \left(\beta_1 + \beta_2 \frac{W}{W_0}\right)$, where

$$\beta_1 = \frac{\frac{\alpha_s}{\alpha_0} - \frac{W_s}{W_0}}{1 - \frac{W_s}{W_0}}; \quad \beta_2 = \frac{1 - \frac{\alpha_s}{\alpha_0}}{1 - \frac{W_s}{W_0}}. \tag{101}$$

Kozusko uses an empirical function of the fat free mass ratio (R), which is defined as fat free weight divided by total body weight, to predict an individual's value of β_2 via the function

$$\beta_2 = \frac{\tanh\left(\frac{f(R-m)}{R*(1-R)}\right) + 1}{2} \tag{102}$$

for parameters f and m to be determined from fitting. The model is compared to Kleiber's scaling law for energy expenditure [39] and the Harris-Benedict equations [40] when applied to data from the Minnesota starvation experiment. The adjustment for reduced energy expenditure allows the model to more accurately capture adaptation leading to lower weight loss, whereas models without this behaviour tend to dramatically overestimate weight lost under caloric restriction. Kozusko's model still overestimates expected weight loss, but is a significant improvement. The free parameters f and m allow fitting to data in order to account for individual variation, which may lead to more accurate prediction for individuals and may thus be of clinical use. The purely empirical basis of this model makes it less informative about mechanisms underlying adaptation; although it describes and may predict individual behaviour, it does not speculate on its physiological basis, nor account for situations with *ad libitum* feeding.

3.7. Antonetti - Energy balance incorporating thermic effect of feeding

Antonetti [41] formulates a similar energy balance model in terms of change of internal energy ΔU, energy from food E_f, energy excreted E_w, environmental heat loss Q_L, and energy expended on work W_k, with a fraction α allocated to specific dynamic action (the increase in heat generation following feeding)

$$\Delta U = (1 - \alpha)(E_f - E_w) - (Q_L + W_k) \tag{103}$$

Acting over a period of days $\Delta\Theta$ and collecting terms $C_D = (E_f - E_w)/\Delta\Theta$, $C = (Q_L + W_k)/\Delta\Theta$ gives the equation

$$\frac{\Delta U}{\Delta\Theta} = (1 - \alpha) C_D - C, \tag{104}$$

which can be expressed as an ODE

$$\frac{dW}{d\Theta} = \frac{(1 - \alpha) C_D - C}{\gamma}, \tag{105}$$

where γ is a constant of proportionality relating energy U to body weight W. Writing Q_L and W_k as scaling in body weight to some power, Antonetti obtains the ODE

$$\frac{dW}{d\Theta} = \frac{(1-\alpha)C_D - (K_A W + K_B W^n)}{\gamma}, \tag{106}$$

which is solved numerically, with the proportionality constants K_A and K_B obtained from data. This yields predictions for body weight change under different activity and calorie intake conditions. Antonetti states that, at the time, clinical data was insufficient to provide a valid test of this equation, although he does attempt to make some comparisons.

3.8. DEB - Generalised energy flow model

Dynamic Energy Budget (DEB) theory attempts to model a generalised organism and its allocation of energy. A review of the basics of DEB theory are presented by Sousa et al [42], which we attempt to summarise here. DEB theory considers the flows of energy in a generalised organism which can invest energy into structure V (which acts as a measure of the 'volume' of the organism), energy reserve E and maturation E_H. Structure and reserve are referred to as 'generalised compounds', which represent many biological compounds making up parts of the organism. Different types of organism are represented by different parametrisations of the model, particularly the parameters representing power allocations to different compartments.

Food intake flow is p_X, of which p_A is assimilated into the reserve and the remainder is excreted. The organism consumes p_C to fuel its activities, so

$$\frac{dE}{dt} = p_A - p_C. \tag{107}$$

Energy flux allocated to growth at rate p_G increases structure V dependent on specific cost of growth E_G

$$\frac{dV}{dt} = \frac{p_G}{E_G}. \tag{108}$$

Energy can also be allocated to somatic maintenance p_M and maturity maintenance p_J. Energy is allocated first in pairs according to an allocation function $\kappa(V, E)$ such that $p_M + p_G = \kappa p_C$ and $p_J + p_R = (1-\kappa)p_C$. Maintenance costs are paid first in this model, with the remainder being allocated to maturity, reproduction, or growth. Feeding is dependent on surface area J_{Xm} and food density X

$$p_X = J_{Xm} V^{2/3} \mu_X f(X), \tag{109}$$

where $f(X)$ scales feeding rate by food availability. Organisms are assumed to have two different 'life-history events', which are signalled by the maturity variable E_H passing set values. These are birth at $E_H = E_H^B$ and puberty at $E_H = E_H^P$. After puberty, maturation stops and no more energy is allocated to maturation. Maturation energy allocation is thus accounted for by the equation

$$\frac{dE_H}{dt} = p_R, \quad E_H < E_H^P. \tag{110}$$

Along with a number of other assumptions, this model can recover a general expression for growth. If abundant food and no change in life stage are assumed, von Bertalanffy's law for the 'size' of the organism L [43] can also be derived

$$\frac{dL}{dt} = r_B(L_\infty - L), \tag{111}$$

where r_B is a function of maintenance rate, food availability, and fraction of energy invested into growth. This is a well-known growth law obeyed by many species. DEB theory has been developed beyond these foundations to account for a wide range of situations and organisms, as is found on the DEB theory website.

3.9. Chow and Hall - Dynamical systems analysis of the Energy Balance model

Chow and Hall investigate the fixed points of a class of energy partition models based around the Energy Balance model [44], [SI 3.2]. They begin with a three compartment flux balance model for fat F, glycogen G, and protein P

$$\rho_F \frac{dF}{dt} = I_F - f_F E \tag{112}$$
$$\rho_G \frac{dG}{dt} = I_C - f_C E \tag{113}$$
$$\rho_P \frac{dP}{dt} = I_P - (1 - f_F - f_F)E, \tag{114}$$

where ρ_F, ρ_G and ρ_P are the energy density of each compartment, I_F, I_C, I_P the intake rates and f_F f_C are the fraction of expenditure derived from each compartment. By assuming that glycogen is constant on long timescales and accounting for the additional mass due to water content of each compartment, this can be rewritten to a two variable model for fat mass F and lean tissue mass L

$$\rho_F \frac{dF}{dt} = I_F - fE \tag{115}$$
$$\rho_L \frac{dL}{dt} = I_L - (1 - f)E. \tag{116}$$

The function $f = f(I_F, I_L, F, L)$ is a function governing how energy is drawn from each compartment to meet expenditure requirements. For instance, imposing Forbes' law [25], [SI 3.1]

$$\frac{dF}{dL} = \frac{F}{10.4} \tag{117}$$

yields the equation

$$f(F, L) = \frac{I_F}{E} - \frac{\alpha}{1+\alpha}\frac{I - E}{E} \tag{118}$$

where $\alpha = \rho_F F/10.4\rho_L$ as before. If intakes I_F and I_L are held constant, the function f will determine the position and nature of the fixed points of the model, which will occur at the

intersection of the F and L nullclines

$$\frac{dF}{dt} = 0 \implies I_F - fE = 0 \tag{119}$$

$$\frac{dL}{dt} = 0 \implies I_L - (1-f)E = 0. \tag{120}$$

A stability analysis of the energy partition model given by Forbes' law reveals the existence of a marginally stable attracting invariant manifold rather than one or more isolated fixed points. This indicates that any temporary change in intake or expenditure will cause body composition to move to a new point on the manifold, rather than return to its previous value before the perturbation. Chow and Hall point out that experiments involving weight change through aerobic exercise or changes in energy intake will be unable to distinguish between different classes of model, and that experiments that directly alter body composition or fat utilisation fraction are necessary to investigate this but have not yet been carried out.

4. Control theoretic models

Models using control theory attempt to find 'optimal' behaviour policies for an organism in response to a given environment. These typically revolve around choosing a policy to maximise some cost function which keeps track of how well the policy deals with the environment. Policies can be deterministic or stochastic, and take place in either discrete or continuous time steps, and these choices determine how the optimal control can be found.

4.1. Davis and Levine - Proportional-integral control of a single feeding bout

Davis and Levine construct a control theoretic negative feedback model for the control of fluid meal size by gut filling based on experimental findings which they review in the introduction to their paper [45]. Intake $I(t)$ is modelled as a proportional-integral controller based on a drinking rate d and an ingestion signal $\epsilon(t)$ such that $I(t) = d\epsilon(t)$. The ingestion signal is taken to have the form:

$$\epsilon(t) = pg(t) - kr\int_0^t I(\tau)d\tau \tag{121}$$

where p is a parameter representing the significance of the food for the animal (for instance providing salty food to a sodium-depleted animal) and $g(t)$ is the concentration of the flavour signalling the relevance of the food. The product gp is referred to as palatability. The parameter r is called the retention coefficient and represents the time taken for fluid to be absorbed into the gut; a small value of r indicates rapid absorption. Finally, k is a proportionality constant that determines how much gut fullness contributes to the decrease in eating rate. Assuming that $g(t)$ is a step function:

$$g(t) = \begin{cases} 0, & t < 0. \\ g, & t \geq 0 \end{cases} \tag{122}$$

the control system can be solved using Laplace transforms to find the ingestion rate $I(t)$

$$I(t) = gpde^{-drkt} \tag{123}$$

and the cumulative ingestion rate $C(t)$

$$C(t) = \int_0^t I(\tau)d\tau \tag{124}$$

$$= \frac{gp}{rk}(1 - e^{-drkt}), \tag{125}$$

which is used to suggest that that palatability plays a crucial role in the total amount of feeding that occurs in a meal. The model shows an ability to fit previous experimental data well over ingestion time ranges of 30 minutes, and can reproduce behaviour from both sham feeding and altered food palatability, but does not predict an ending time for the feeding bout.

4.2. Tam et al. - Leptin-mediated control of body weight

Tam et al. [46] consider leptin-mediated regulation of energy homeostasis on a timescale of weeks in order to distinguish between set-point and settling-point control of bodyweight, and to investigate the effects of leptin resistance. Leptin production into the bloodstream (which has volume V) is assumed to be proportional to fat mass F with a synthesis rate R_{syn} and is removed from the blood by the kidneys in a concentration-dependent manner with rate R_{clear}. Plasma leptin L_p enters the brain through both saturable and non-saturable pathways, leading to changes in brain leptin L_b. Thus for both set-point and settling-point models the leptin equations are (with some condensing of notation)

$$\frac{d(L_pV)}{dt} = R_{syn}F - R_{clear}L_p \tag{126}$$

$$L_b = k_1\frac{L_p}{k_2 + L_p} + k_3L_p. \tag{127}$$

The rate constants R_{syn}, R_{clear}, k_1, k_2, and k_3 are all derived from literature values rather than fitted. Body mass M is given by fat mass F and lean mass M_L, with changes in body mass occurring solely due to increases in fat mass. Fat mass changes according to the imbalance between intake I and expenditure E, leading to the body composition equations

$$M = F + M_L \tag{128}$$

$$\frac{dF}{dt} = \frac{1}{\rho_F}(E - I) \tag{129}$$

where ρ_F is the energy density of fat.

In the settling-point model energy expenditure E and intake I are given by saturable functions of brain leptin concentration

$$I = k_4\left(1 - \frac{L_b}{k_5 + L_b}\right) \tag{130}$$

$$E = k_6M\left(1 + k_7\frac{L_b}{k_8 + L_b}\right). \tag{131}$$

Rate constants k_4, k_6 and k_7 are derived from literature values, with k_5 and k_8 fitted from data.

The set-point model describes energy intake and expenditure through the use of a proportional-integral controller which scales E and I to return L_b to a set point L_b^*

$$I = a_1(L_b - L_b^*) + a_2 \int_0^t (L_b - L_b^*)dt + c_1 \tag{132}$$

$$E = M(a_3(L_b - L_b^*) + a_4 \int_0^t (L_b - L_b^*)dt + c_2). \tag{133}$$

Constants c_1 and c_2 are derived from intake and expenditure at steady-state in the settling-point model. a_1 - a_4 could not be fitted directly from data, so were chosen arbitrarily to prevent oscillations, however they do not affect the steady-state values of the set-point model.

Leptin resistance was modelled by changing the value of the rate constant k_2, which modifies the blood-brain transport of leptin. Due to a lack of experimental data, the authors used the following phenomenological equation

$$\hat{k}_2(L_p) = k_2 + k_9(L_p - k_{10})\Theta(L_p - k_{10}) \tag{134}$$

using the Heaviside step function Θ, k_9 is a scaling factor and k_{10} is the leptin concentration where resistance begins.

Tam et al.propose a combination model which incorporates features of both the set point and settling-point model, as neither fully account for observed experimental data; the set-point model is too effective in preventing obesity, whereas the settling-point model does not provide a strong enough defence of body weight.

4.3. Jacquier et al. - Endocrine control of body composition usng the Energy Balance model

Jacquier et al. [47] construct a model of coupled ODEs linking energy balance Δ_E to changes in food intake and energy expenditure, with food intake being controlled by variables representing leptin, glucose, and insulin. Energy expenditure is determined by fat mass S and fat-free mass W, as well as an activity parameter ξ and rate parameter R. Food intake is given as the minimum of hunger h and available food a, giving an energy balance equation

$$\Delta_E = \min(a, h) - R(\rho_W W + \rho_S S + \xi) \tag{135}$$

where ρ_S and ρ_W are energy densities of fat and fat-free tissue respectively. The evolution equations for S and W are

$$\frac{dS}{dt} = \frac{\Delta_E}{\rho_W x + \rho_S}; \quad \frac{dW}{dt} = \frac{\Delta_E x}{\rho_W x + \rho_S} \tag{136}$$

as in the Hall model, $x = dW/dS = \zeta + \psi \exp(\kappa S)$ governs the partitioning of energy between fat and lean mass. In this case the parameters are estimated during model fitting. Plasma leptin l is given by:

$$\frac{dl}{dt} = \gamma_2 S - \gamma_1 l, \tag{137}$$

with production rate γ_2 and clearance rate γ_1. Plasma glucose u is governed by a similar equation involving food intake, production rate μ_1 and clearance rate μ_2:

$$\frac{du}{dt} = \mu_1 \min(a, h) - \mu_2 u \tag{138}$$

Ghrelin e is inhibited by food intake, and has the following rate equation:

$$\frac{de}{dt} = \frac{\nu_2}{1 + \nu_1 \min(a, h)} - \nu_3 e. \tag{139}$$

These three variables are combined to determine the hunger variable h, given in terms of number of Joules required

$$\frac{dh}{dt} = \frac{\alpha_1 e}{1 + \alpha_2 l} - \beta(\alpha_3 + u)h. \tag{140}$$

Allowing hunger to depend on endocrine levels, which in turn depend on metabolic state, makes this model more physiologically relevant, and considers food intake as a feedback control system. Finally, the rate parameter R is allowed to vary to simulate adaptation to different food intakes by comparing food consumed in a recent period to that eaten over a long time period

$$\frac{dR}{dt} = \epsilon \left(\frac{1}{\tau} \int_{t-\tau}^{t} \min(a(v), h(v)dv - \frac{1}{\tau'} \int_{t-\tau'}^{t} \min(a(v), h(v)dv \right). \tag{141}$$

6 of the 21 parameters were obtained from the literature, with 12 obtained from model fitting, and the remainder from experiments and data regarding diet. Parameters governing combination of endocrine signals is determined entirely from fitting. The model predicts consumption and body weight behaviour well under a variety of scenarios, including scenarios the model was not fitted to. Adaptation (in terms of varying R) is shown to be important in recapitulating experimental observations.

4.4. Booth et al. - Multiple-factor control of rat feeding

Booth et al.formulated a computational closed-loop feedback control model for feeding behaviour at the level of individual meals [48, 49]. The main strength of this model is that although it simulates down to a fine time resolution, it also models long-timescale dynamics by allowing fat reserves to modulate feeding behaviour. In addition it incorporates circadian effects and a simple model for energy expenditure due to time-varying metabolic rate, as well as stochastic feeding behaviour. These features make it probably the most fully-realised model for energy homeostasis developed to date, however as it predates many modern endocrinological discoveries it is not formulated in terms of specific endocrine mechanisms. The model is formulated as a block diagram, which we reproduce below (Figure 1), before outlining a number of the most important dynamical equations in the model. The central regulator of feeding in the Booth model is the energy flow E, which is comprised of absorption of energy from the gut A, flow to/from lipids L, metabolic rate M and the gut satiety E_V

$$E = A - L - M - E_V. \tag{142}$$

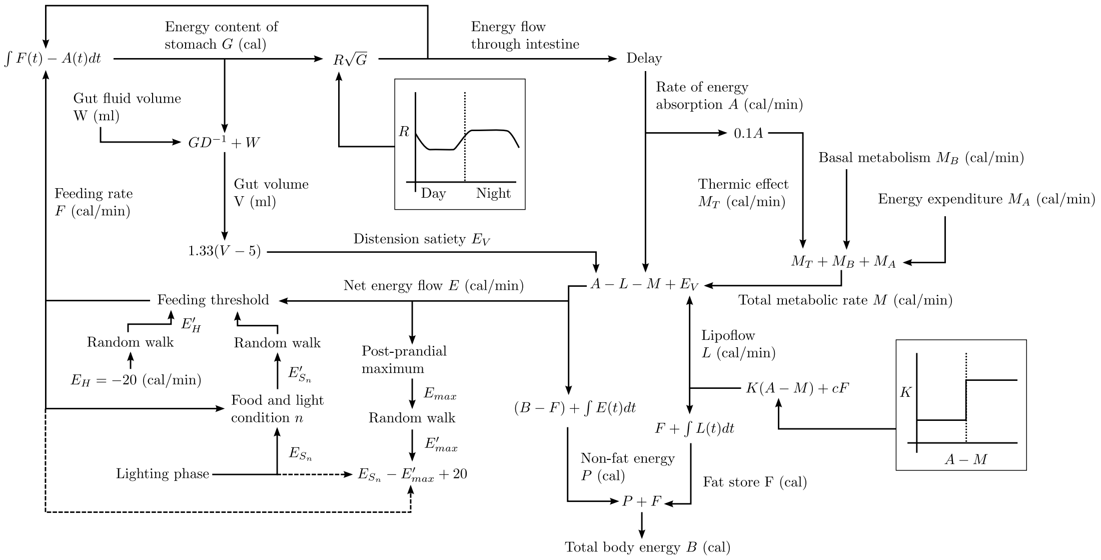

Figure 1: Block diagram for the Booth energy flow model. Important equations are described in more detail in the text below. Based on a figure from from [49]

Energy flow triggers feeding (or its cessation) through a two-threshold model; if $F = 0$ and energy flow is less than some threshold E'_H then feeding rate becomes some positive constant F. Feeding then continues until a satiety threshold E'_S is reached, at which point $F = 0$. These thresholds can be a constant, however Booth et al.also consider thresholds undergoing a mean-reverting random walk. Food enters the stomach, the fullness of which depends on feeding rate F as well as absorption $A(t)$

$$\frac{dG}{dt} = F(t) - A(t). \tag{143}$$

The absorption rate into the bloodstream depends on a varying rate $R(t)$ and the energy content of the stomach G

$$A(t) = R(t)\sqrt{G(t)}. \tag{144}$$

A number of other factors such as the thermic effect of feeding and sensory qualities of food are included in the model (see Figure 1). Simulating the model generates predictions for feeding bouts, metabolic rates, and body mass, and can reproduce experimental results to a reasonable degree of accuracy [50].

4.5. McFarland and Sibly - A general framework for optimal behaviour

McFarland and Sibly [51, 52] attempt to formulate behaviour in terms of an optimality principle. By denoting all environmental and physiological factors relevant to the behaviour under consideration as a vector $\mathbf{x}$ (which is referred to as causal factor space) and denoting behaviour sequences as movement within causal factor space, given some cost function

$C(\mathbf{x}, \mathbf{u})$ the fitness of a behaviour sequence $u(t)$ over a time interval $[0, T]$ is defined as being

$$F[\mathbf{u}(t)] = \int_0^T C(\mathbf{x}, \mathbf{u}) dt. \quad (145)$$

The Pontryagin minimum principle gives the optimal behaviour sequence by maximising a Hamiltonian-like equation:

$$H = \sum_{i=1}^{N} p_i \dot{x}_i - C \quad (146)$$

$$\dot{p}_i = \frac{\partial C}{\partial x_i}, \quad (147)$$

where N is the dimension of causal factor space. This can be solved analytically for quadratic cost functions

$$C = \sum_{i=1}^{N} x_i^2 + u_i^2 \quad (148)$$

$$\dot{p}_i = \frac{\partial C}{\partial x_i} = 2x_i, \quad (149)$$

which yields an exponentially decaying intake rate and an exponentially satiating cumulative intake - the same prediction as Davis and Levine's control theory based model. This indicates that their mechanistic approach, which agreed well with behavioural observations, is also optimal according to this cost function.

4.6. McNamara and Houston - Dynamic programming for optimal behaviour

McNamara and Houston [53] consider a behavioural sequence over a finite interval $[0, T]$, discretised into time periods $t = 0, 1, 2, \ldots, T$. This behaviour sequence is typically assumed to be a short period of time with no opportunity for reproduction in $[0, T]$. The fitness of a behaviour sequence is indicated by how beneficial it is to the organism's chance of reproduction at the end of the time interval, which is denoted $R(x)$ for state x. Actions can have stochastic payoffs, so the state at time t is a random variable denoted X_t, with x representing a definite state in a discrete state space. Policies (series of actions a) are denoted by π. Because both time and state are discretised, there is a finite set of combinations of x and t, and this problem is solvable for the optimal policy using dynamic programming. The central object of this approach is the following function

$$\psi^*(x, t) = \max_{\pi} \mathbb{E}_{\pi}[R(X_T | X_t = x)], \quad (150)$$

which gives the expected future reproductive success from (x, t) if the organism follows the optimal policy. This can be shown to provide a way to choose the optimal action a^* for a given (x, t)

$$H(x, a, t) = \mathbb{E}_a[\psi^*(X_{t+1}, t + 1) | X_t = x] \quad (151)$$

$$\psi^* = \max_a H(x, a, t) \quad (152)$$

which also allows a way of calculating the cost of performing a suboptimal action a'

$$c(x, a', t) = H(x, a^*, t) - H(x, a', t). \tag{153}$$

This framework is applied to an example of a small bird that can perform one of several foraging actions at several time points during the day, or rest and expend no energy. The bird expends energy overnight and cannot forage, so must end the day with enough energy to survive. The dynamic programming approach allows the calculation of optimal policies for this problem. By using the equation for the cost of a suboptimal policy McNamara and Houston offer an explanation for the 'dawn chorus': it is typically less costly to perform a non-foraging task in the first time period of the day given a relatively small level of reserves, as it is likely that foraging for the rest of the day will be sufficient to allow survival.

4.7. Niyogi et al. - Optimal stochastic policies in working for a reward

Niyogi et al.formulate a model of mouse behaviour at the level of individual activity bouts, motivated by a brain stimulation reward [54]. Although not directly modelling feeding behaviour, this model has the potential to be adapted to this end as both feeding and work for stimulation reward are settings in which some activity (feeding, lever pressing) is carried out for some reward (food, brain stimulation). The main difference is that brain stimulation does not appear to 'satiate', whereas the utility of food decreases significantly as more is eaten. Their model is formulated in economic terms; brain stimulation reward and leisure are both modelled as possessing some level of utility. A bout of brain stimulation reward has utility RI, and leisure time has a reward which varies as a function of leisure time duration $C_L(\tau)$ for leisure time τ. Niyogi et al.investigate reward functions that are linear, supralinear, and weighted combinations of linear and supralinear in τ. In order to obtain the reward, subjects are required to work for a duration called the 'price' P. Work in the pre-reward state consists of bouts of work interspersed with bouts of leisure. Once the accumulated work time w reaches P, brain stimulation reward is administered. Following the reward there is a further leisure bout of length $\tau_{\mathrm{Pav}} + \tau_L$ (see [54] for details on the origin of the term τ_{Pav}) before the model transitions to the pre-reward state again. The state space S is therefore a combination of a discrete component in $\{pre, post\}$ and a continuous component $w \in [0, P)$, and as such has some similarities to a class of stochastic models known as piecewise deterministic Markov processes [55, 56]. The subject chooses between actions depending on a stochastic policy π, which determines the total average reward rate ρ^π

$$\rho^\pi = \frac{RI + \mathbb{E}_{\pi([L,\tau_L]|\mathrm{post}))}[C_L(\tau_{Pav} + \tau_L)] + \int_0^P \mathbb{E}_{\pi_{w_L}}[\sum_{n_{L|[pre,w]}} C_L(\tau_L)]dw}{P + \mathbb{E}_{\pi([L,\tau_L]|\mathrm{post}))}[\tau_L] + \tau_{Pav} + \int_0^P \mathbb{E}_{\pi_{w_L}}[\sum_{n_{L|[pre,w]}} \tau_L]dw}, \tag{154}$$

where $\pi([L, \tau_L]|post)$ and π_{w_L} are the probabilities of engaging in leisure for a time τ_L in the post- and pre-reward states respectively, and $n_{L|[pre,w]}$ is the (random) number of leisure periods in the pre-reward interval. The policy π for a given action and its duration is state-dependent, and is assumed to be a soft-max policy, so greater expected returns will

be preferred but suboptimal choices are sometimes made. The softmax policy equation for a subject in state s is

$$\pi([a,\tau_a]|s) = \frac{\exp\left[\beta Q^\pi(s,[a,\tau_a])\right]\mu_a(\tau_a)}{\sum_{a'}\int_{\tau_{a'}}\exp\left[\beta Q^\pi(s,[a',\tau_{a'}])\right]\mu_{a'}(\tau_{a'})d\tau_{a'}}, \tag{155}$$

for reward values $Q^\pi(s,[a,\tau_a])$ of engaging in action a for duration τ_a given current state s. The free parameter β specifies the degree of stochasticity, and the function $\mu_a(\tau_a)$ is a prior over durations. Value functions for leisure in the pre- and post-reward states are

$$Q^\pi(pre,[L,\tau_L]) = C_L(\tau_L) - \rho^\pi\tau_L + V^\pi([pre,w]) \tag{156}$$
$$Q^\pi(post,[L,\tau_L]) = C_L(\tau_L+\tau_{Pav}) - \rho^\pi(\tau_L+\tau_{Pav}) + V^\pi([pre,0]) \tag{157}$$

where the three terms represent the utility of leisure, the opportunity cost of leisure (given by the average reward value), and the long-run value of transitioning to the following state V^π. This is given by the equation

$$V^\pi(s) = \sum_a\int_{\tau_a}\pi([a,\tau_a]|s)Q^\pi(s,[a,\tau_a]). \tag{158}$$

The Q-function for working in the pre-reward state is more complex as there are two possible outcomes: accumulated work is sufficient to earn reward, or it is not. These are represented by the first and second term respectively in the following equation

$$Q^\pi([pre,w],[W,\tau_W]) = \mathbb{1}(w+\tau_W < P)[-\rho^\pi\tau_W + V^\pi([pre,w+\tau_W])] \tag{159}$$
$$+\,\mathbb{1}(w+\tau_W \geq P)[RI - \rho^\pi(P-w) + V^\pi(post)]. \tag{160}$$

Niyogi et al.consider low, medium, and high-payoff situations, and find that in the high payoff setting subjects work as much as possible, whereas in low-payoff situations almost no work is done. In the medium payoff case the model predicts multiple work bouts with short leisure bouts, followed by a leisure bout of duration greater than the experiment's length.

4.8. Yamaguchi et al. - Learning rewards from behaviour

Yamaguchi et al. formulate a reinforcement learning (RL) model of thermotactic behaviour in *C. elegans* [57] based on maximum entropy inverse reinforcement learning [58]. In RL individuals act to maximise reward, which they accmplish by trading off exploration (in order to find rewarding states) and exploitation (using the high-reward states already discovered). In inverse RL, we assume that these rewards have already been completely discovered by the agent, which is acting to maximise these rewards. We do not know the agent's reward function, however, and seek to determine it from data. An important tool for this is the value function $V(s)$, which maps states s to a number indicating the long-term reward expected from that state. For an agent with some decision-making policy π that maps states to actions (which we will return to later), the value of a state s under π is given by

$$V^\pi(s) = \mathbb{E}[R|s,\pi] \tag{161}$$

where R is the sum of future rewards r_t, discounted at some rate $\gamma \in [0, 1]$

$$R = \sum_{t=0}^{\infty} \gamma^t r_t. \tag{162}$$

The aim of RL is to find the optimal policy π^* that maximises the return. We will not go into methods for doing so, however an excellent introductory textbook is available [59]. In order to infer r, we first infer V, but to do so, we need a model of the agent's decision policy. Yamaguchi et al. model thermotaxis as having a preference for both temperature T and its derivative dT, meaning that climbing or descending the temperature gradient as appropriate is explicitly rewarded. Worms are modelled as having both 'passive' stochastic dynamics $p(s'|s)$ that give the uncontrolled motion of the worms as well as the decision-making behaviour given by π. Passive dynamics captures both inertial motion and 'diffusive' motion, given by

$$P((T', dT')|(T, dT)) = \mathcal{N}(T'|T + dT\delta t, \sigma_T)\mathcal{N}(dT'|dT, \sigma_{dT}), \tag{163}$$

where $\mathcal{N}(x|\mu, \sigma)$ is the likelihood of x from a normal distribution of mean μ and variance σ. Under the assumption that rewards are penalised by their divergence from the passive dynamics, the policy π^* is given by

$$\pi^*(s'|s) = \frac{P(s'|s)\exp(-v(s')}{\sum_{s'} P(s'|s)\exp(-v(s'))}, \tag{164}$$

which gives the likelihood of the observed state transitions $s_t \to s_{t+1}$:

$$L = \Pi_t \pi^*(s_{t+1}|s_t). \tag{165}$$

This allows inference of the value function (and thus rewards) given observations of behaviour. They find inverse RL can recover synthetic data correctly, and also learn meaningful rewards and values from observed data.

4.9. Fulcher, Phillips, and Robinson - Neural mass theoretic treatment of sleep/wake cycle quantitatively predicts behaviour

Neural mass theory is a simple ODE model of neuronal dynamics in response to external stimuli. Each distinct neuronal population (for example monoaminergic neurons) is treated as a single variable, which represents the average firing rate of that population. When two neuronal populations are connected, the mean cell body potential of the downstream population changes as a sigmoid function of the firing rate of the upstream population. External stimuli are represented as drives altering the mean cell body potential of each population directly. Fulcher, Phillips, and Robinson [60] build on a previously developed homeostatic model of sleep regulation due to the interaction between the mutually inhibitory wake-promoting monoaminergic (MA) and sleep-promoting ventrolateral preoptic nuclues (VLPO) neuronal populations [61]. In neural mass theory the firing of each population of

cells is averaged out into a single mean firing rate, denoted Q_M for MA cells and Q_V for VLPO cells. The firing rate Q of a population of cells is a sigmoidal function of the mean cell-body potential of the population V

$$Q(V) = \frac{Q_{max}}{1 + exp\{-(V - \theta)/sigma\}}, \tag{166}$$

where Q_{max} is the maximum firing rate, θ is the mean firing threshold relative to resting, and $\sigma\pi/\sqrt{3}$ is the standard deviation of the firing rate distribution of the population. All of these quantities are in principle measurable from electrophysiological experiments, and may be measurable by two-photon imaging techniques. The two populations inhibit one another and respond to exogenous drives D_V and D_M according to standard neural mass dynamics (as reviewed in [62])

$$\tau_V \frac{dV}{dt} = \nu_{VM} Q_M + D_V \tag{167}$$

$$\tau_M \frac{dM}{dt} = \nu_{MV} Q_V + D_M \tag{168}$$

$$. \tag{169}$$

The rate of response for a given neuronal population is τ_j and ν_{jk} is the strength of the effect of populations k on populatio j, which can be negative (inhibitory) or positive (excitatory). Fulcher, Philips, and Robinson explore the effects of a homeostatic and circadian sleep drives H and C on VLPO as well as a constant drive A on MA, as well as impulsive drives ΔD_V, ΔD_M such as loud noises. Drives to VLPO and MA are given by

$$D_V = \nu_{VC} C + \nu_{VH} H + \Delta D_V \tag{170}$$

$$D_M = \nu_{MA} A + \Delta D_M. \tag{171}$$

The dynamics of C and H are given by

$$\chi \frac{dH}{dt} = \mu Q_M - H \tag{172}$$

$$C(t) = \sin(\omega t) + c_0, \tag{173}$$

where χ is the clearance rate of the somnogen and μ gives the rate of somnogen production when MA neurons are firing (indicating an awake state). C is circadian, so $\omega = (2\pi/24)h^{-1}$. Comparison to experimental data reveals that the model predicts the level of auditory stimulus required to wake sleeping subjects given the time since sleep onset. The similarity of this system to the AGRP/POMC feeding control circuit is striking; a mutually inhibitory pair of neurons with by homeostatic and circadian drives, whose dynamics in turn cause behaviour that regulates the homeostatic state. It is possible that an application of neural mass theory to the AGRP/POMC system may also yield quantiative predictions of behaviour.

References

[1] Bergman RN, Phillips LS, Cobelli C. Physiologic evaluation of factors controlling glucose tolerance in man: measurement of insulin sensitivity and beta-cell glucose sensitivity from the response to intravenous glucose. Journal of Clinical Investigation. 1981;68(6):1456.

[2] De Gaetano A, Arino O. Mathematical modelling of the intravenous glucose tolerance test. Journal of Mathematical Biology. 2000;40(2):136–168.

[3] Bolie VW. Coefficients of normal blood glucose regulation. Journal of Applied Physiology. 1961;16(5):783–788.

[4] Li J, Kuang Y, Mason CC. Modeling the glucose–insulin regulatory system and ultradian insulin secretory oscillations with two explicit time delays. Journal of Theoretical Biology. 2006;242(3):722–735.

[5] Li J, Kuang Y, Li B. Analysis of IVGTT glucose-insulin interaction models with time delay. Discrete and Continuous Dynamical Systems Series B. 2001;1(1):103–124.

[6] Roy A, Parker RS. Dynamic modeling of free fatty acid, glucose, and insulin: An extended "minimal model". Diabetes Technology & Therapeutics. 2006;8(6):617–626.

[7] Akaike H. A new look at the statistical model identification. IEEE Transactions on Automatic Control. 1974;19(6):716–723.

[8] Dalla Man C, Camilleri M, Cobelli C. A system model of oral glucose absorption: validation on gold standard data. IEEE Transactions on Biomedical Engineering. 2006;53(12):2472–2478.

[9] Lehmann E, Deutsch T. A physiological model of glucose-insulin interaction in type 1 diabetes mellitus. Journal of biomedical engineering. 1992;14(3):235–242.

[10] Elashoff JD, Reedy TJ, Meyer JH, et al. Analysis of gastric emptying data. Gastroenterology. 1982;83(6):1306–1312.

[11] Siegel J, Urbain J, Adler L, Charkes N, Maurer A, Krevsky B, et al. Biphasic nature of gastric emptying. Gut. 1988;29(1):85–89.

[12] Goodfellow I, Bengio Y, Courville A. Deep learning. MIT Press; 2016.

[13] Hamilton JD. Time series analysis. vol. 2. Princeton University Press; 1994.

[14] Shumway RH, Stoffer DS. Time series analysis and its applications: with R examples. Springer Science & Business Media; 2006.

[15] Cryer JD, Chan KS. Time Series Analysis. Springer New York; 2008.

[16] Drucker H, Burges CJ, Kaufman L, Smola AJ, Vapnik V. Support vector regression machines. In: Advances in neural information processing systems; 1997. p. 155–161.

[17] Smola AJ, Schölkopf B. A tutorial on support vector regression. Statistics and computing. 2004;14(3):199–222.

[18] Topp B, Promislow K, Devries G, Miura RM, T Finegood D. A model of β-cell mass, insulin, and glucose kinetics: Pathways to diabetes. Journal of Theoretical Biology. 2000;206(4):605–619.

[19] Wang YF, Khan M, van den Berg HA. Interaction of fast and slow dynamics in endocrine control systems with an application to β-cell dynamics. Mathematical Biosciences. 2012;235(1):8–18.

[20] Sturis J, Polonsky KS, Mosekilde E, Van Cauter E. Computer model for mechanisms underlying ultradian oscillations of insulin and glucose. American Journal of Physiology-Endocrinology And Metabolism. 1991;260(5):E801–E809.

[21] Tolić IM, Mosekilde E, Sturis J. Modeling the insulin–glucose feedback system: the significance of pulsatile insulin secretion. Journal of Theoretical Biology. 2000;207(3):361–375.

[22] Polonsky K, Given B, Van Cauter E. Twenty-four-hour profiles and pulsatile patterns of insulin secretion in normal and obese subjects. Journal of Clinical Investigation. 1988;81(2):442.

[23] Shapiro ET, Tillil H, Polonsky KS, Fang VS, Rubenstein AH, Cauter EV. Oscillations in Insulin Secretion During Constant Glucose Infusion in Normal Man: Relationship to Changes in Plasma Glucose. The Journal of Clinical Endocrinology & Metabolism. 1988;67(2):307–314.

[24] Verdonk CA, Rizza RA, Gerich JE. Effects of plasma glucose concentration on glucose utilization and glucose clearance in normal man. Diabetes. 1981;30(6):535–537.

[25] Forbes GB. Lean body mass-body fat interrelationships in humans. Nutrition Reviews. 1987;45(10):225–231.

[26] Hall KD. Body fat and fat-free mass inter-relationships: Forbes's theory revisited. British Journal of Nutrition. 2007;97(06):1059–1063.

[27] Hall KD, Sacks G, Chandramohan D, Chow CC, Wang YC, Gortmaker SL, et al. Quantification of the effect of energy imbalance on bodyweight. The Lancet. 2011;378(9793):826–837.

[28] Hall KD. Metabolism of mice and men: mathematical modeling of body weight dynamics. Current Opinion in Clinical Nutrition & Metabolic Care. 2012;15(5):418–423.

[29] Hall KD. Modeling Metabolic Adaptations and Energy Regulation in Humans. Annual Review of Nutrition. 2012;32:35–54.

[30] Guo J, Hall KD. Estimating the continuous-time dynamics of energy and fat metabolism in mice. PLoS Comput Biol. 2009;5(9):e1000511.

[31] Guo J, Hall KD. Predicting changes of body weight, body fat, energy expenditure and metabolic fuel selection in C57BL/6 mice. PLoS ONE. 2011;6(1):e15961.

[32] Alpert SS. Growth, thermogenesis, and hyperphagia. The American Journal of Clinical Nutrition. 1990;52(5):784–792.

[33] Payne PR, Dugdale AE. A model for the prediction of energy balance and body weight. Annals of Human Biology. 1977;4(6):525–535.

[34] Dugdale A, Payne P. Pattern of lean and fat deposition in adults. Nature. 1977;266(5600):349–351.

[35] Speakman JR. A nonadaptive scenario explaining the genetic predisposition to obesity: the predation release hypothesis. Cell Metabolism. 2007;6(1):5–12.

[36] Speakman JR, Levitsky DA, Allison DB, Bray MS, de Castro JM, Clegg DJ, et al. Set points, settling points and some alternative models: theoretical options to understand how genes and environments combine to regulate body adiposity. Disease Models and Mechanisms. 2011;4(6):733–745.

[37] Speakman JR, Westerterp KR. A mathematical model of weight loss under total starvation: evidence against the thrifty-gene hypothesis. Disease Models and Mechanisms. 2013;6(1):236–251.

[38] Kozusko FP. Body weight setpoint, metabolic adaption and human starvation. Bulletin of Mathematical Biology. 2001;63(2):393–403.

[39] Kleiber M. Body size and metabolic rate. Physiological Reviews. 1947;27(4):511–541.

[40] Harris JA, Benedict FG. A biometric study of human basal metabolism. Proceedings of the National Academy of Sciences. 1918;4(12):370–373.

[41] Antonetti VW. The equations governing weight change in human beings. The American Journal of Clinical Nutrition. 1973;26(1):64–71.

[42] Sousa T, Domingos T, Kooijman S. From empirical patterns to theory: a formal metabolic theory of life. Philosophical Transactions of the Royal Society of London B: Biological Sciences. 2008;363(1502):2453–2464.

[43] Von Bertalanffy L. Quantitative laws in metabolism and growth. Quarterly Review of Biology. 1957;p. 217–231.

[44] Chow CC, Hall KD. The dynamics of human body weight change. PLoS Computational Biology. 2008;4(3):e1000045.

[45] Davis JD, Levine MW. A model for the control of ingestion. Psychological Review. 1977;84(4):379.

[46] Tam J, Fukumura D, Jain RK. A mathematical model of murine metabolic regulation by leptin: energy balance and defense of a stable body weight. Cell Metabolism. 2009;9(1):52–63.

[47] Jacquier M, Crauste F, Soulage CO, Soula HA. A predictive model of the dynamics of body weight and food intake in rats submitted to caloric restrictions. PloS ONE. 2014;9(6):e100073.

[48] Booth D, Toates F, Platt S. Control system for hunger and its implications in animal and man. Hunger; Basic Mechanisms and Clinical Implications, D Novin, W Wyrwicka & GA Bray, eds. 1976;.

[49] Booth D. Prediction of feeding behaviour from energy flows in the rat. Hunger Models: computable theory of feeding control. 1978;p. 227–278.

[50] Guillot A, Meyer JA. A test of the Booth energy flow model (Mark 3) on feeding patterns of mice. Appetite. 1987;8(1):67–78.

[51] McFarland D, Sibly R. The behavioural final common path. Philosophical Transactions of the Royal Society of London B: Biological Sciences. 1975;270(907):265–293.

[52] Sibly R, McFarland D. On the fitness of behavior sequences. The American Naturalist. 1976;110(974):601–617.

[53] McNamara JM, Houston AI. The common currency for behavioral decisions. The American Naturalist. 1986;127(3):358–378.

[54] Niyogi RK, Breton YA, Solomon RB, Conover K, Shizgal P, Dayan P. Optimal indolence: a normative microscopic approach to work and leisure. Journal of The Royal Society Interface. 2014;11(91):20130969.

[55] Davis MH. Piecewise-deterministic Markov processes: A general class of non-diffusion stochastic models. Journal of the Royal Statistical Society Series B (Methodological). 1984;p. 353–388.

[56] Davis MH. Markov Models & Optimization. vol. 49. CRC Press; 1993.

[57] Yamaguchi S, Naoki H, Ikeda M, Tsukada Y, Nakano S, Mori I, et al. Identification Of Animal Behavioral Strategy By Inverse Reinforcement Learning˜ Its Application To Thermotaxis In C. elegans˜. bioRxiv. 2017;p. 129007.

[58] Ziebart BD, Maas AL, Bagnell JA, Dey AK. Maximum Entropy Inverse Reinforcement Learning. In: AAAI. vol. 8. Chicago, IL, USA; 2008. p. 1433–1438.

[59] Sutton RS, Barto AG. Reinforcement learning: An introduction. vol. 1. MIT Press; 1998.

[60] Fulcher B, Phillips A, Robinson P. Modeling the impact of impulsive stimuli on sleep-wake dynamics. Physical Review E. 2008;78(5):051920.

[61] Phillips A, Robinson P. A quantitative model of sleep-wake dynamics based on the physiology of the brainstem ascending arousal system. Journal of Biological Rhythms. 2007;22(2):167–179.

[62] Deco G, Jirsa VK, Robinson PA, Breakspear M, Friston K. The dynamic brain: from spiking neurons to neural masses and cortical fields. PLoS Computational Biology. 2008;4(8):e1000092.
